# *In Silico* Screening of Isocitrate Lyase for Novel Anti-Buruli Ulcer Natural Products Originating from Africa

**DOI:** 10.3390/molecules23071550

**Published:** 2018-06-27

**Authors:** Samuel K. Kwofie, Bismark Dankwa, Emmanuel A. Odame, Francis E. Agamah, Lady P. A. Doe, Joshua Teye, Odame Agyapong, Whelton A. Miller, Lydia Mosi, Michael D. Wilson

**Affiliations:** 1 Department of Biomedical Engineering, School of Engineering Sciences, College of Basic and Applied Sciences, University of Ghana, P. O. Box LG 77, Legon, Accra, Ghana; dankwabismark52@gmail.com (B.D.); kayaddo3@gmail.com (E.A.O.); francisagamahh@gmail.com (F.E.A.); agbanupaula@gmail.com (L.P.A.D.); jteye@st.ug.edu.gh (J.T.); oagyapomg@gmail.com (O.A.); 2 Department of Biochemistry, Cell and Molecular Biology, West African Center for Cell Biology and Infectious Pathogens, University of Ghana, P. O. Box LG 77, Legon, Accra, Ghana; lmosi@ug.edu.gh; 3 Department of Parasitology, Noguchi Memorial Institute for Medical Research (NMIMR), College of Health Sciences (CHS), University of Ghana, P. O. Box LG 77, Legon, Accra, Ghana; Mwilson@noguchi.ug.edu.gh; 4 Department of Chemical and Biomolecular Engineering, School of Engineering and Applied Science, University of Pennsylvania, Philadelphia, PA 19104, USA; wheltonm@seas.upenn.edu; 5 Department of Chemistry & Physics, College of Science and Technology, Lincoln University, Philadelphia, PA 19104, USA

**Keywords:** virtual screening, homology modeling, buruli ulcer, *Mycobacterium ulcerans*, molecular dynamics, natural product

## Abstract

Buruli ulcer (BU) is caused by *Mycobacterium ulcerans* and is predominant in both tropical and subtropical regions. The neglected debilitating disease is characterized by chronic necrotizing skin lesions attributed to a mycolactone, which is a macrolide toxin secreted by *M. ulcerans*. The preferred treatment is surgical excision of the lesions followed by a prolonged combination antibiotic therapy using existing drugs such as rifampicin and streptomycin or clarithromycin. These antibiotics appear not to be adequately potent and efficacious against persistent and late stage ulcers. In addition, emerging drug resistance to treatment poses great challenges. There is a need to identify novel natural product-derived lead compounds, which are potent and efficacious for the treatment of Buruli ulcer. Natural products present a rich diversity of chemical compounds with proven activity against various infectious diseases, and therefore, are considered in this study. This study sought to computationally predict natural product-derived lead compounds with the potential to be developed further into potent drugs with better therapeutic efficacy than the existing anti-buruli ulcer compounds. The three-dimensional (3D) structure of Isocitrate lyase (ICL) of *Mycobacterium ulcerans* was generated using homology modeling and was further scrutinized with molecular dynamics simulations. A library consisting of 885 compounds retrieved from the AfroDb database was virtually screened against the validated ICL model using AutoDock Vina. AfroDb is a compendium of “drug-like” and structurally diverse 3D structures of natural products originating from different geographical regions in Africa. The molecular docking with the ICL model was validated by computing a Receiver Operating Characteristic (ROC) curve with a reasonably good Area Under the Curve (AUC) value of 0.89375. Twenty hit compounds, which docked firmly within the active site pocket of the ICL receptor, were assessed via *in silico* bioactivity and pharmacological profiling. The three compounds, which emerged as potential novel leads, comprise ZINC38143792 (Euscaphic acid), ZINC95485880, and ZINC95486305 with reasonable binding energies (high affinity) of −8.6, −8.6, and −8.8 kcal/mol, respectively. Euscaphic acid has been reported to show minimal inhibition against a drug-sensitive strain of *M. tuberculosis*. The other two leads were both predicted to possess dermatological activity while one was antibacterial. The leads have shown promising results pertaining to efficacy, toxicity, pharmacokinetic, and safety. These leads can be experimentally characterized to assess their anti-mycobacterial activity and their scaffolds may serve as rich skeletons for developing anti-buruli ulcer drugs.

## 1. Introduction

Buruli ulcer (BU) is a devastating chronic skin infection caused by *Mycobacterium ulcerans* [1,2]. It is the third most common mycobacterial disease after tuberculosis and leprosy [3]. The disease is characterized by necrotizing skin lesions, which can cover up to a third of the entire body surface area of the affected individual. It can also lead to irreversible deformity or long-term functional disability when not properly treated [2].

Although its mode of transmission remains poorly understood [4,5,6], studies have shown that it occurs in communities where there are slow flowing rivers, streams and water bodies [6]. Azumah et al. have also demonstrated the involvement of *Acanthamoeba* in the disease transmission and progression in animal experiments [7,8]. Although the disease occurs elsewhere, the majority of cases are in sub-Saharan Africa where, for example, 1676 of the 1864 new cases of Buruli ulcer worldwide in 2016 were reported from this region [9].

The World Health Organization (WHO) recommends the use of the combination regimen of either rifampicin and streptomycin or rifampicin and clarithromycin administered for eight weeks as the first-line of treatment of BU cases [7,10,11,12]. However, long-term administration of streptomycin has side effects that includes damage to the cochlea and vestibulum [11]. Streptomycin can be substituted with clarithromycin or moxifloxacin, which are milder. There is currently no substitute for rifampicin [13,14,15]. Therefore, any resistance to it may render this treatment strategy ineffective [16]. Furthermore, the long duration of the treatment regimen also engenders non-compliance among patients who reside mostly in remote communities that are distant from health centers. In its severe form, the sole recourse is surgery. The procedure can be performed only at facilities with adequate medical infrastructure and are not affordable for most patients [17].

The current treatment options are very limited. However, there are a few studies being conducted to mitigate it including amikacin administered in combination with rifampicin and azithromycin, which has been found to have antimicrobial activity against *M. ulcerans* in-vitro [17]. Results from a 2017 controlled pilot-study to access the efficacy of a combination of rifampicin and streptomycin or rifampicin and clarithromycin highlighted no significant difference in their curative rates [18]. Therefore, there is an urgent need for new drugs that combine efficacy with minimal side effects at low costs [19]. The conventional methods for discovery of new drugs are time-consuming, laborious, and very expensive and an alternative approach that can overcome these challenges is the use of computer-aided structure-based drug design (SBDD) [4,20]. Talele et al. have highlighted successful drugs such as Captopril (Capoten^®^, Bristol Myers-Squibb, New York, NY, USA), Saquinavir (Invirase^®^, Hoffmann-La Roche, Basel, Switzerland) and Zanamivir (Relenza^®^, Gilead Sciences, Foster City, CA, USA) that have been developed using this strategy [21]. SBDD methods have also been employed to study the inhibitors of *Mycobacterium tuberculosis* [22,23,24].

The key enzymes of mycobacterium’s glyoxylate shunt are isocitrate lyase (ICL) and malate synthase (MS). This metabolic pathway enables the organism to make direct use of the intermediates of the tricarboxylic acid (TCA) cycle for gluconeogenesis and other biosynthetic processes essential for the survival and persistence of *M. tuberculosis* in macrophages [25]. The protein sequence of ICL contains neutrophilic cysteine residues located in a flexible loop, which undergoes extensive conformational changes after binding to isocitrate (substrate). This results in the complete closure of the active sites. These changes disrupt the pathway, hence, the identification of the ICL as the target of anti-MTb drug discovery studies. The ICL of MTb has a close homologue in *M. ulcerans*, which then offers the opportunity to perform similar studies for the latter organism. Moreover, the ICL gene is not found in mammals. Therefore, in principle, it is not expected to cause any safety concerns using drugs that target it [26,27]. However, unexpected effects resulting from probable drug off-targets [28,29,30] cannot be entirely ruled out.

The attributes of targeting ICL are that: (i) it is essential in the pathogenesis of the disease; (ii) it has no known homologous protein in humans; (iii) it plays a key role in the glyoxylate cycle, which is an essential metabolic pathway; and (iv) the availability of three dimensional (3D) structural data of homologues for use as templates for modelling protein targets [31]. Moreover, ICL has been the target for drug discovery in several studies [27].

The overall goal of the study was to screen ligands from African flora, which are known to contain diverse natural products with scaffolds and various physicochemical properties [32]. In addition, biologically active natural products have the unique abilities to effect specific changes in disease-related metabolic pathways that result in better therapeutic responses [33].

The specific aims were to predict, with a high degree of accuracy, the three dimensional (3D) structure of the *M. ulcerans* ICL receptor, perform molecular dynamics simulation of the model, and virtually screen natural products of African origin against ICL model to identify drug leads [34]. In addition, investigate the mechanism of binding between the ICL and ligands as well as pharmacologically profile the leads using *in silico* techniques to identify compounds with potential anti-mycobacterial activity.

## 2. Results and Discussion

### 2.1. Homology Modeling

The protein sequence of isocitrate lyase from *M. ulcerans* retrieved from the NCBI database had the GenBank accession number EUA86150.1 and comprised 428 amino acid residues [35]. The query sequence was compared to all sequences of known structures stored in Protein Data Bank [36,37] (PDB) via a Basic Logical Alignment and Search Tool (BLAST) search [38,39], which generated a list of protein structures that were similar to the query sequence. To corroborate the results, another template query using SWISS-MODEL template search interface (https://swissmodel.expasy.org/interactive) was performed [40]. Both template search platforms gave the same results. The chain A of the structures were considered for the study. Templates considered were isocitrate lyase from *Mycobacterium tuberculosis* with PDB codes of 5DQL, 1F8I, 1F8M, and 1F61 and percentage sequence identities of 91.12%, 91.10%, 91.10%, and 90.87%, respectively. They were considered for homology modeling because they had higher sequence identity when compared to the other templates. All four templates comprising 5DQL, 1F8I, 1F8M, and 1F61 were solved using X-ray crystallography with resolutions of 1.8, 2.0, 1.8, and 2.2 Å, respectively.

The structural and sequence similarity between the templates (5DQL, 1F8M, 1F61, and 1F8I) were assessed by calculating their multiple sequence alignment using the malign command in Modeller 9v17 [41] and further structural alignments of the four three-dimensional (3D) structures used malign3d command in Modeller 9v17.

**Figure 1:**
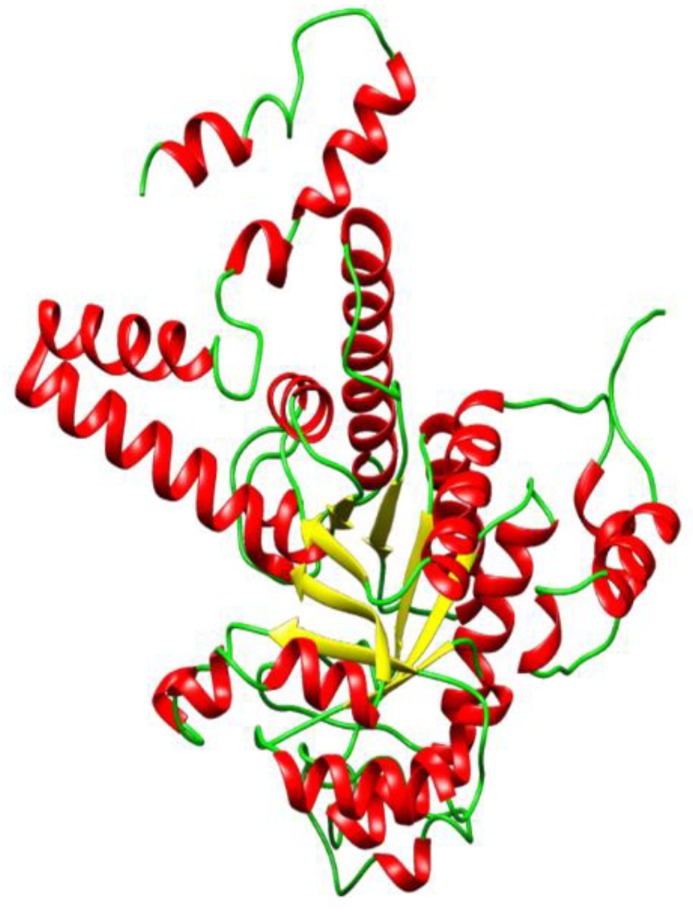
Cartoon representation of the predicted 3D structure of Isocitrate lyase of the *Mycobacterium ulcerans*. The alpha helices are shown in red, the beta sheets are shown in yellow, and the loops are shown in green.

The 3D structure of ICL from *M. tuberculosis* with PDB ID 5DQL was obtained as the most plausible template for homology modeling because it had a slightly higher sequence identity of 91.12% when compared to the others despite having the same resolution of 1.8 Å with 1F8M. Five homology models were generated using Modeller 9v17 and model 5 (Figure 1) was chosen as the best based on the least Discrete Optimized Potential Energy (DOPE) score of −47291.21875 (Table 1). DOPE is an atomic distance-dependent statistical potential that is used to determine the native states of proteins. Therefore, it gives an account of the finite and spherical shape of the native structures [42,43,44].

ModRefiner [45,46] was then used to refine the protein model before carrying out molecular dynamics simulation in GROMACS [47,48,49]. The refined protein had residue Glu155 in the disallowed region. Loop refinement of the protein was then performed using the Modloop server [50] to move the residue back into allowed regions of the protein.

### 2.2. Structure Validation and Quality Prediction

**Figure 2:**
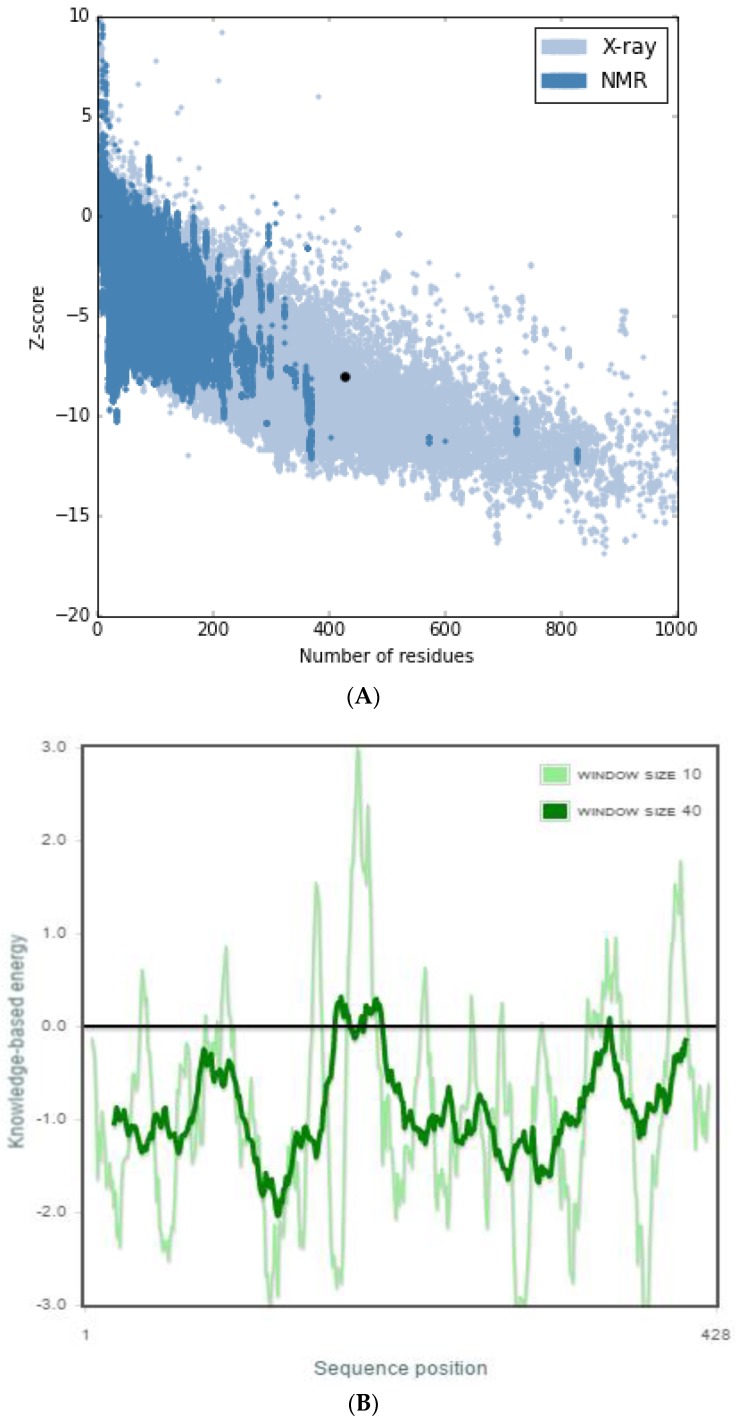
ProSA web z-score and energy graph of ICL modeled protein. (**A**) The Z-score of ICL (represented in dot) was present in the range of all protein chains in the Protein Data Bank determined by X-ray crystallography and nuclear magnetic resonance spectroscopy with respect to their sequence length. (**B**) Energy plot of the ICL protein model.

**Figure 3:**
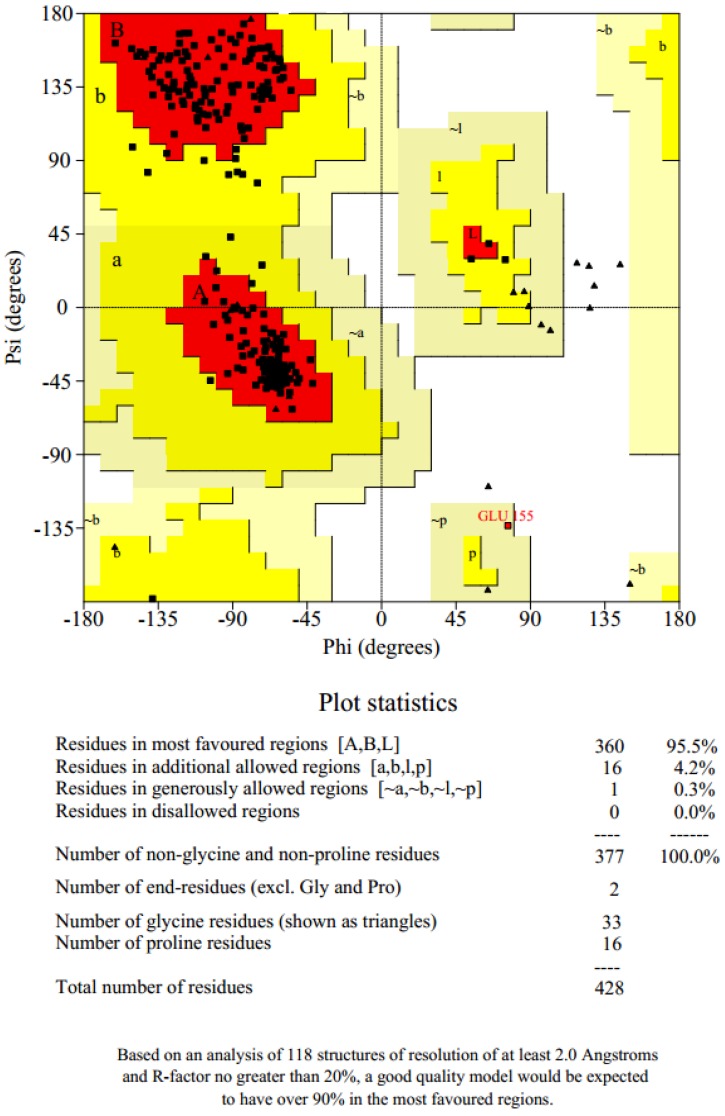
Ramachandran plot of ICL protein structure. This plot provides the general overview of the allowed and disallowed regions of the torsional angle values of the model. Protein with over 90% of its residues in favored regions indicates a model of reasonably high quality.

PROSA [51] is a quality measure tool that compares the overall model quality score of the protein to that of experimentally solved protein structures in the PDB database, which was used to validate the protein model and the results were displayed in a plot (Figure 2a). A z-score of −8.0 was obtained, which showed that the modeled protein falls within the range of X-rays solved protein structures. A more negative z-score implies a better protein model [52]. The local model quality of the protein was also generated (Figure 2b). Amino acids residues with more negative energy levels have a high tendency of contributing to the overall quality of the tertiary structure. The protein was also submitted to the ProQ server to predict protein quality based on its LGscore [53] and MaxSub score, which are its quality measures. MaxSub is a quality measure calculated from the largest number of residues that can be found in which all distances between the model and the correct structure are shorter than 3.5 Å [53]. Likewise, the LGscore is a *p*-value score for the significance of a structural similarity match. The protein had an LGscore of 3.690 and a MaxSub score of 0.296, which falls in the range of a “very good model” per prediction ranges [54]. The stereochemical quality of the refined protein was checked using the Ramachandran plot, which was generated with PROCHECK [55]. The Ramachandran plots of the modeled protein structure before and after loop refinement were generated. The Ramachandran plot drawn before loop refinement placed 95.5% of residues in allowed regions with 0.3% of them in disallowed region while, after loop refinement, 95.5% of residues were placed in allowed regions with no residues in disallowed regions (Figure 3). This implies that the loop refinement process was successful as Glu155, which was initially within the disallowed regions, was successfully placed in the allowed regions of the protein. The secondary structure of the protein model is composed of 17 helices and 6 strands.

### 2.3. Molecular Dynamics Simulations

**Figure 4:**
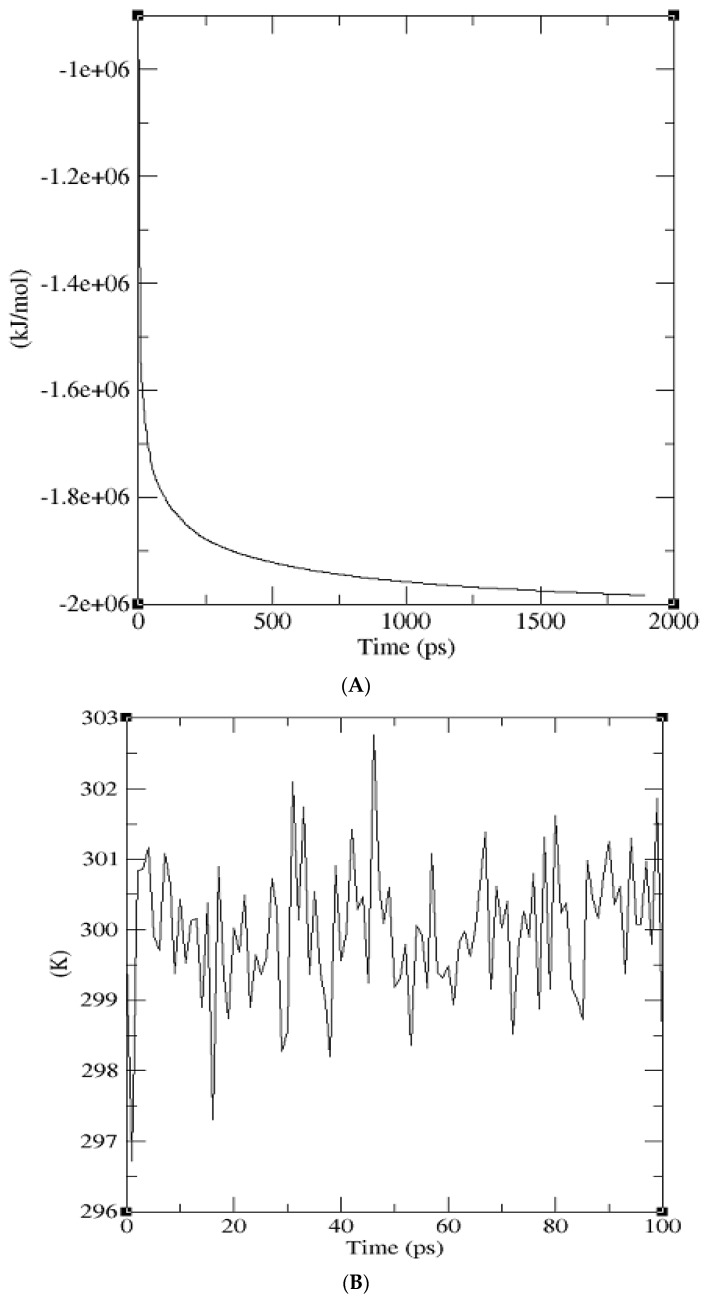

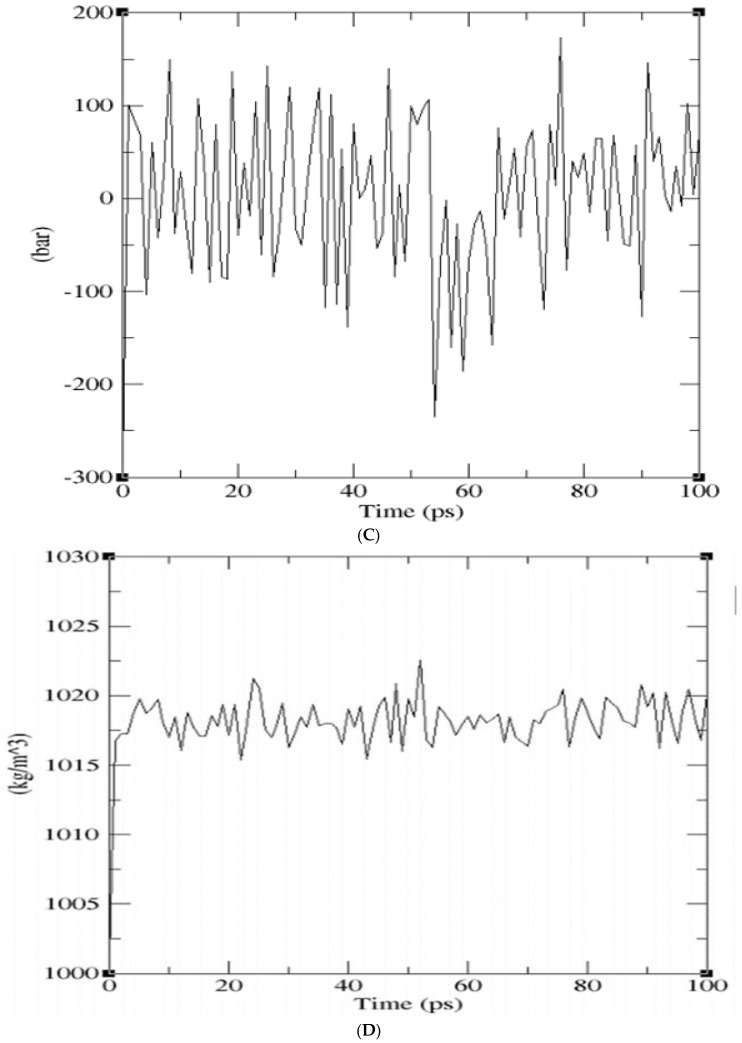

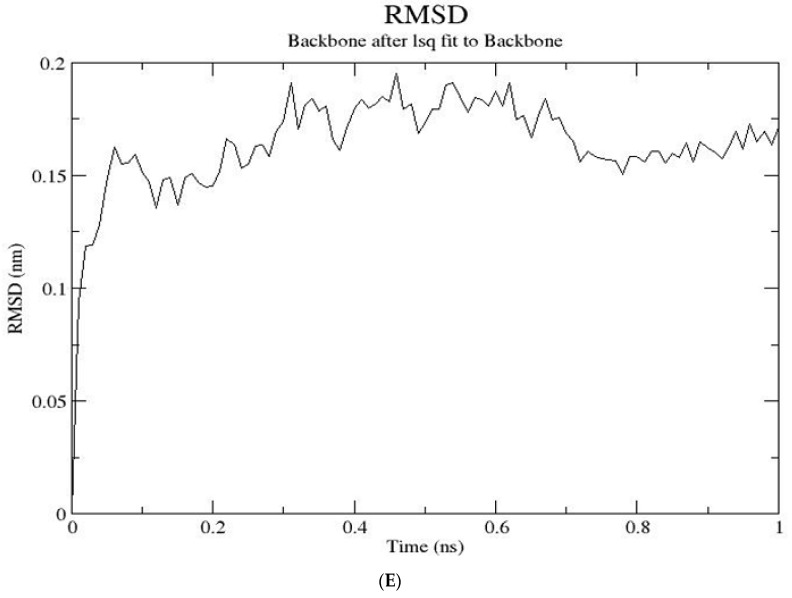
Graphs generated from molecular dynamics simulation. (**A**) Potential energy against time graph of the energy minimized protein produced from GROMACS. The overall potential energy of the model achieved after simulation was −1.9786255e+06 kcal/mol. (**B**) Temperature against time graph showing that the protein temperature was simulated within 300 K. (**C**) A graph of pressure against the time of the simulated protein model. The pressure laid within 1 bar over the period of 100 ps. (**D**) Density against the time graph of the protein after simulation led to an average density of 1018.14 kg/m^3^. (**E**) RMSD graph with deviation stabilizing around 1.6 Å at the end of 1 ns production run.

GROMACS 5.1.1 [47,48,49] was used to minimize the energy of the protein until it reached stability. The minimization was done using the steepest descent method for 1647 steps and the production run was performed for 1 ns. The overall potential energy observed was −1.9786255e+06 kcal/mol where the maximum force converged to 8.9850818e+02 kcal/mol, which is less than the allowable tolerance of 1000 kcal/mol that was set prior to simulation (Figure 4a). Both the NVT and NPT ensemble were run for 100 ps. An average temperature of 300 K was obtained after the 100 ps equilibration phase, which is shown in the temperature graph (Figure 4b). The pressure graph generated showed that the pressure fluctuated widely over the course of the 100 ps equilibration phase. During equilibration, the average value of the pressure was 1.09 bar (Figure 4c). A plot of density against time was generated at the end of isothermal-isobaric ensemble and the running average density was recorded to be 1018.14 kg/m^3^ (Figure 4d). The values obtained for the density variation over 100 ps remained stable over time, which indicates that the system was well equilibrated. The RMSD graph shows a sharp increase in the deviation starting from around 0.2 Å to about 1.9 Å and then stabilizes around 1.6 Å within 1000 ps.

### 2.4. Active Site Detection

**Figure 5:**
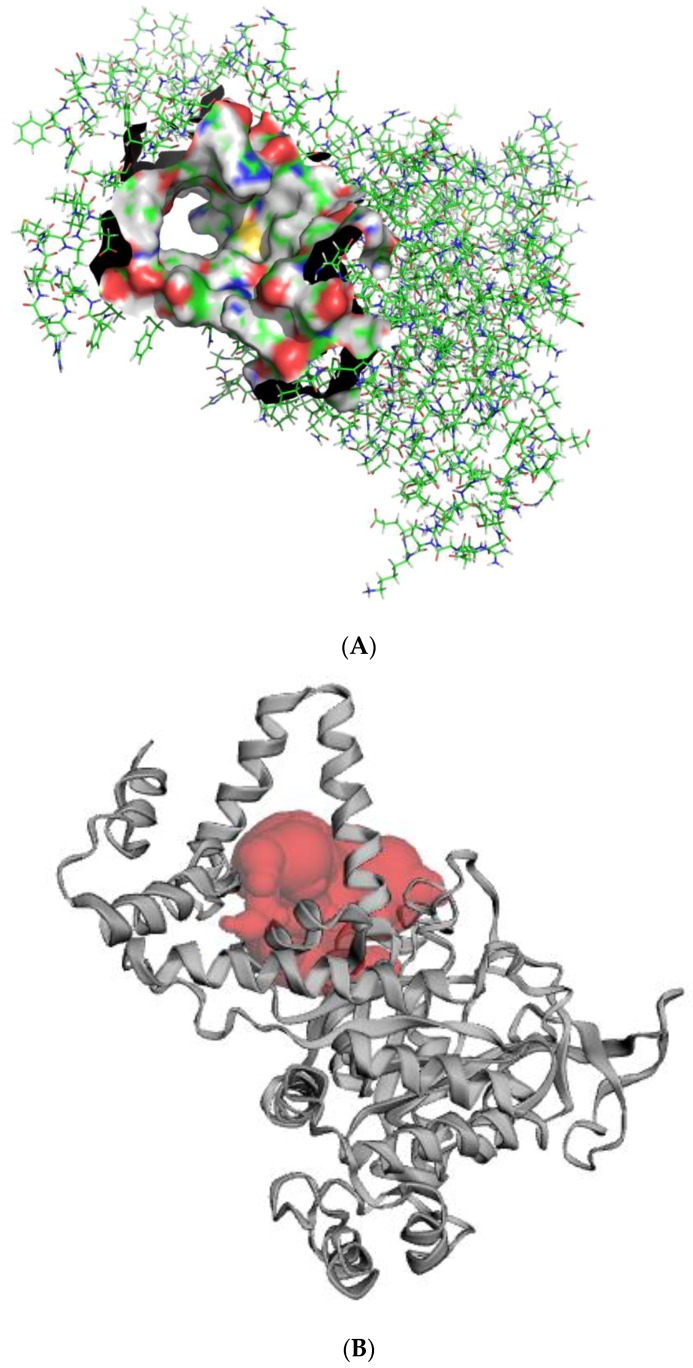
Representation of the predicted active site of the protein model. (**A**) The entire protein model is represented in lines with the surface representation constituting the active site. (**B**) Cartoon representation of the protein model and the active site. The red shows the substrate binding region.

CASTp predicted 78 binding pockets as both solvent accessible and molecular surfaces. The active site was chosen based on the pocket with the largest volume and surface area, which were 1502.289 nm^3^ and 962.759 nm^2^, respectively. The site comprised 54 residues, which included Asn67, Leu69, Thr73, Gly74, Asn75, Met76, Val78, Gln79, Gln80, Arg82, Ala83, Leu85, Ala127, Arg130, Ala131, Ile134, Pro289, Cys314, Ser315, Pro316, Phe318, Asn319, Trp320, Lys321, Leu324, Asp325, Asp326, Ile329, Ala330, Lys331, Phe332, Gln333, Ile346, Leu348, Ala349, Gly350, His352, Ala353, Leu354, Tyr356, Ser357, Asp360, Leu361, Leu376, Arg379, Glu380, Ala383, Arg386, Tyr388, Ala390, Thr391, His393, Glu396 and Val397 (Figure 5).

### 2.5. Virtual Screening Library of Natural Products

Molecular docking is most frequently used in SBDD because it has the ability to predict, with a substantial degree of accuracy, the conformation of small-molecules (ligands) within the appropriate target binding site [56]. The AutoDock Vina search space center was set to spatial coordinates of 49.7731, 52.376, and 72.30 Å in the X, Y, and Z coordinate axes. The dimensions of the grid box were set to 59.9038, 66.8920, and 44.779 Å by taking into account the entire search space of the protein molecule in order to perform docking. The ligand protein complexes were visualized in PyMOL to identify ligands, which docked firmly within the active site pocket and also had high negative binding energy values. The top 100 hit compounds were visualized after the virtual screening process. Each of the hundred compounds had their binding energies less than or equal to −8.4 kcal/mol with the lowest binding energy being −10.5 kcal/mol. The more negative the values of the binding energy, the better the predicted binding affinity between the ligand and the target. The binding energy values of the virtual screening results were measured as kcal/mol. After visualization of the pose of hundred ligands within the active site, 20 of the ligands were observed to dock firmly and deeply within the active site. Their LigPlots showed the individual residues interacting with the ligands via hydrogen bonding and hydrophobic interactions (Table 2 and Table S2).

### 2.6. Protein-Ligand Interactions

Hydrogen bond interactions between the ligand and the protein were studied via the Ligplot of the ligand-protein complexes using Ligplot+ and PyMOL. A total of 20 selected ligands of the 100 top hits were observed to dock properly upon visualization after careful study. Hydrogen bonding and hydrophobic interactions are weak intermolecular forces that play key roles in stabilizing ligands energetically at favorable regions of a protein structure [57]. After visualization of pose, the 20 selected hits compounds that formed hydrogen and hydrophobic bond interactions with the residues of the active site of the receptor are shown in Table 2 and Table S2. Each of the ligands had several hydrophobic interactions with a majority of residues within the active site. In terms of hydrogen bonding, ZINC95486006, ZINC95486007, ZINC38143792, and ZINC95485880 had the highest number of hydrogen bond interactions. ZINC95486006 had four hydrogen bonds, and therefore, interacted with residues Asn75, Ser357, Glu380, and Ala390. Similarly, ZINC95486007 had three hydrogen bonds with residues Glu380, Asn75, and Ala390. ZINC38143792 also formed three hydrogen bonds with Arg379, Glu380, and Ser357. Finally, ZINC95485880 also formed three strong hydrogen bond interactions with Glu380, Arg386, and His393. The bond length of all hydrogen bond interactions were less than 5 Å. Upon careful observation, each of these four ligands formed hydrogen bonding with Glu380. Two other ligands formed hydrogen bond interactions with Glu380. Likewise, Asn75 was also involved in the hydrogen bond interactions of six of the ligands. Additionally, Glu380 and Asn75 could be essential residues in the active site of the protein (Table 2 and Table S2). ZINC95486305, ZINC95486303, and ZINC95485905 had two hydrogen bonds interactions each with the receptor. ZINC95485882 was the only top ligand that did not have any hydrogen bond interactions with any of the residues despite its good binding energy, and therefore, was ranked to the bottom of the Table (Table 2 and Table S2). Each of the remaining ligands had one hydrogen bond interaction with only one residue in the active site except ZINC95486001, which had two hydrogen bond interactions with the same residue (Asn 75).

### 2.7. Docking Protocol Validation

#### 2.7.1. Superimposition and Alignment

**Figure 6:**
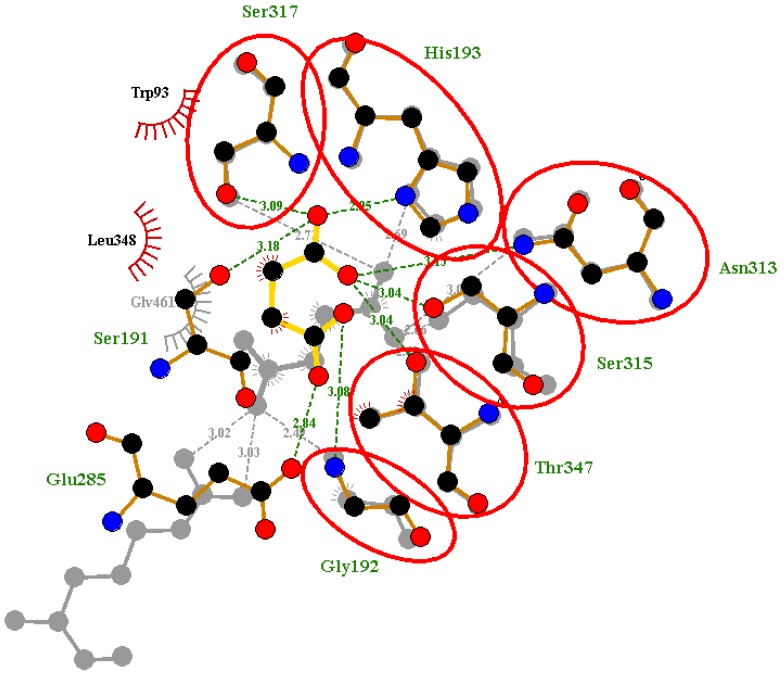
Superimposed Ligplots comparing the interactions between the co-crystalized ligand of 1F8I and the re-docked Succinic ligand. Residues circled in red represent the overlapped molecular interactions of both the co-crystalized ligands and the re-docked complex.

**Figure 7:**
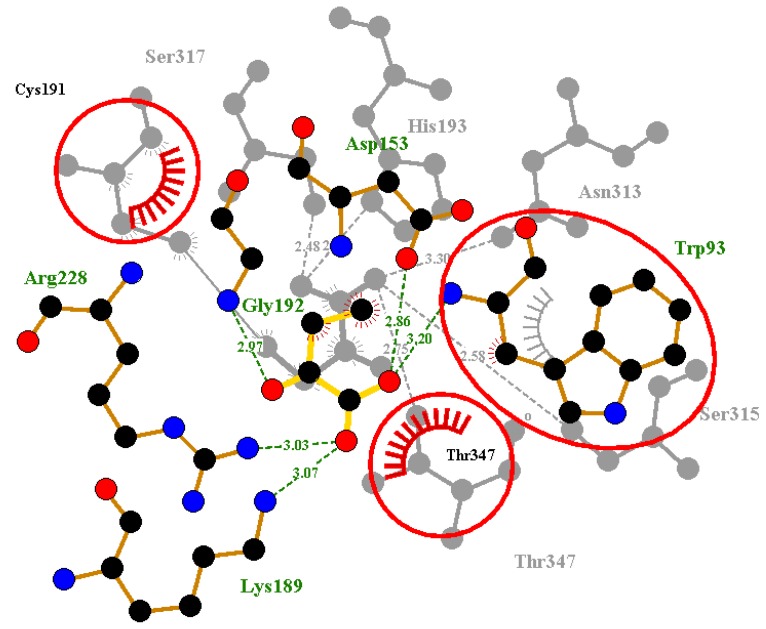
Superimposed Ligplots comparing the interactions between the co-crystalized ligand of 5DQL and the re-docked 4-hydroxy-2-oxobutanoic acid ligand. Residues circled in red represented the predicted molecular interaction of the co-crystalized and re-docked complex.

After re-docking and superimposition, the predicted docking poses and the experimentally determined poses of the co-crystallized ligands upon alignment shared common interactions with some specific residues in the active site. However, superimposition of the crystallographic ligand and redocking pose was used as a means of validating docking [58]. We superimposed the co-crystal complex and the re-docked ligand complex in order to identify critical overlapping residues. The overall goal still remained unchanged since we were able to reasonably validate AutoDock4 using the re-docking method. When the re-docked pose of Succinic acid of 1F8I was superimposed with that of the co-crystalized ligand, there was overlap of six critical residues with their corresponding hydrogen bond interactions (Figure 6). These residues include Gly192, His193, Asn313, Ser315, Ser317 and Thr347. This shows that AutoDock4 was able to virtually reproduce a similar pose in the same environment we performed the virtual screening. Likewise, for the predicted pose of pyruvic acid of 1F8M receptor, there was overlap of five critical residues with their corresponding hydrogen and hydrophobic bond interactions. The overlapped residues comprise Trp93, Cys191, His193, Asn313 and Ser315 (Figure S1). In addition, for the 5DQL used as a template for the structural modeling, the re-docked pose of 4-hydroxy-2-oxobutanoic acid ligand was superimposed with the co-crystalized ligand. There was an overlap of critical residues comprising Trp93, Cys191, and Thr347 with their corresponding hydrogen and hydrophobic bond interactions (Figure 7). Among the ligands, only glyoxylic acid of 1F8I formed overlapping molecular interactions with two of the critical residues comprising Arg228 and Thr347 upon re-docking and subsequent superimposition (Figure S2). Upon docking the four ligands to the generated ICL model of *M. ulcerans*, only pyruvic acid had one overlapping interaction involving Asn313 upon comparing with the co-crystalized structure (PDB ID 1F8M) (Figure S3). Even though, the other three ligands formed hydrogen and hydrophobic bond interactions with residues within the selected active site, no overlapping interactions with their corresponding residues were observed upon superimposition with their co-crystalized ligands. They, nonetheless, had binding energies below −5 kcal/mol. The binding energies for the ligands comprising 4-hydroxy-2-oxobutanoic acid, glyoxylic acid, succinic acid, and pyruvic acid are −4.0, −4.5, −4.9, and −4.6 kcal/mol, respectively. Furthermore, the validation of the molecular docking was also undertaken by aligning the re-docked ligands with their respective co-crystalized complexes [59]. The RMSD values of the alignment of the re-docked ligands with the co-crystalized complexes of 1F8I, 1F8M, and 5DQL are 1.801 Å, 1.218 Å, and 1.769 Å, respectively. All the RMSD values of the alignments are well below 2.0 Å, which is considered the threshold for good alignment [60].

#### 2.7.2. ROC Curve Analysis

**Figure 8:**
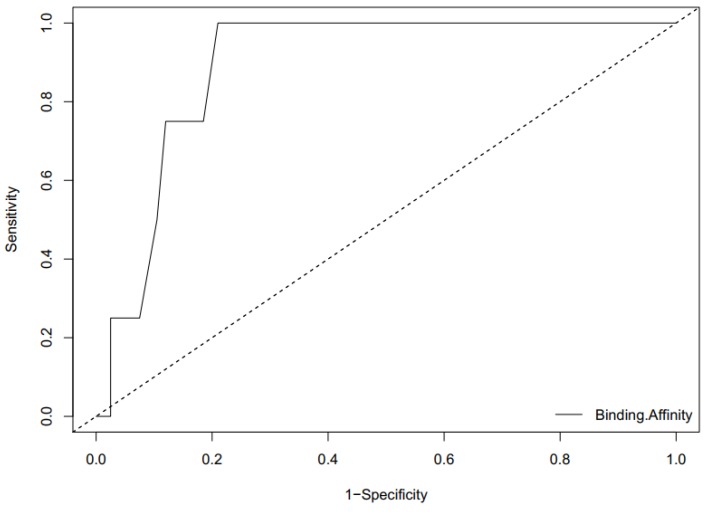
An ROC curve generated by screening co-crystalized ligands from ICL of *M. tuberculosis* with corresponding decoys against the model structure of ICL of *M. ulcerans*. The AUC of the ROC curve is 0.89375, which is considered reasonably good.

Area Under the Curve (AUC) of the Receiver Operating Characteristic (ROC) is a plausible metric for evaluating the classification ability of a docking model to distinguish between docked decoys and active ligands. The ROC curve provides a graphical representation of the overall performance of the docking to discriminate amongst the active ligands and the decoys when screened against the ICL receptors [61,62,63]. When AUC of ROC is closer to 1, the higher the ability of the model to discriminate between active ligands and decoys and closer to 0 is an indication of poor classification. AUC of 1 means perfect classification between active ligands and decoys with the system able to distinguish between true and false cases without errors. The value of 0.5 implies poor prediction ability with average random selection, and less than 0.70 is indicative of moderate discrimination [62,64]. The values of the AUC of the ROC curve for the active ligands and the 199 decoys screened separately against the model of ICL of *M. ulcerans*, 1F8I, 1F8M, and 5DQL are 0.89375, 07625, 0.76938, and 0.73567, respectively (Figure 8 and Figures S4–S6). AUC values from 0.8 to 0.9 are considered to be reasonably good while 0.7 to 0.8 are acceptable [65,66].

### 2.8. Pharmacological Studies for Discovery of Leads

After virtual screening, the 20 selected hit compounds and 5 known drugs comprising rifampicin, streptomycin, clarithromycin, moxifloxacin, and amikacin were subjected to ADME/Tox studies and physicochemically profiled using Lipinski’s rule of five (molecular weight not more than 500 Da, hydrogen bond donor not more than 5, hydrogen bond acceptors not more than 10, and log-*p* value not greater than 5) [67,68]. Upon prediction via SwissADME [69], eight of the top compounds and four known drugs were observed to have violated two or more of Lipinski’s rule (Table 3 and Table S3). In addition, 12 out of the 20 top compounds were predicted as either water insoluble or poorly soluble (Table 3 and Table S3). Out of the five drugs, rifampicin was predicted to be poorly soluble while amikacin was predicted to be soluble. This property, therefore, implied that the majority of the predicted hit compounds may exhibit poor oral administration (Table 3 and Table S3). It was also observed that five of the predicted top compounds comprising ZINC38143792, ZINC95485880, ZINC95486231, ZINC95485943, and ZINC03941105 complied with all ADMET filtering rules including that of Lipinski’s. Among these five compounds, ZINC38143792 and ZINC95485880 were considered as leads because aside complying with all Lipinski rules, these compounds also had satisfactory solubility properties. Furthermore, they were not inhibitors to any of the Cytochrome P450 isoenzymes, which implies sufficient drug elimination properties via metabolic biotransformation, and therefore, should be given more priority (Table 4). ZINC38143792 was also predicted to be a non-Blood Brain Barrier Permeant (BBB) (Table 4). However, ZINC95485880 failed and we can suggest that these compounds could likely not interfere with the activities of the nervous system by permeating or crossing boundaries of the blood brain barrier. While some compounds violated a rule of Lipinski, they, nonetheless, did very well in other ADMET properties such as solubility, bioavailability score, gastrointestinal absorption, and toxicity. Prominent among them was ZINC95486305, which was then added to the list of probable leads. The essence of toxicity profiling was to determine the cardiac toxicity and mutagenicity using ADMET predictor (Version 8.1). Cardiac toxicity was based on the hERG model, which predicts whether the compound blocks the hERG K+ channel or not. A “yes” indicate a compound has the likelihood to block the channel and a “no” indicate otherwise. From Table 5, ZINC95486182 and ZINC95486142 failed the toxicity tests while all the others passed. None of the compounds were predicted to be mutagenic (Table 5).

### 2.9. Prediction of Lead Compounds

Generally, discovering lead compounds after virtual screening is based on three primary criteria which are binding energy, molecular bond interactions and pharmacological profiling. Lead compounds are the most probable compounds, which have very low binding energies, strong hydrogen and hydrophobic bond interactions as well as reasonably good ADMET properties. Therefore, after virtual screening of the AfroDb database, 20 top compounds were selected as promising candidates for further analysis based on their low binding energies and reasonably good poses within the active site pocket. Out of this number, ZINC95486006 and ZINC95486007 formed four and three hydrogen bonds, respectively, within the active site but both had unfavorable ADMET properties. Irrespective of their strong hydrogen bond interactions, these ligands had very high molecular weights of 666.805 Da and 668.821 Da. In addition, each possessed 12 hydrogen bond acceptors which violated the Lipinski’s rule of five. ZINC95486183, ZINC95486184, ZINC95486142, ZINC95486182, and ZINC95486303 also formed hydrogen bond interactions, but their ADMET properties were nonetheless very poor and fell short in molecular weight and log-*p* value of the Lipinski’s rule.

**Figure 9:**
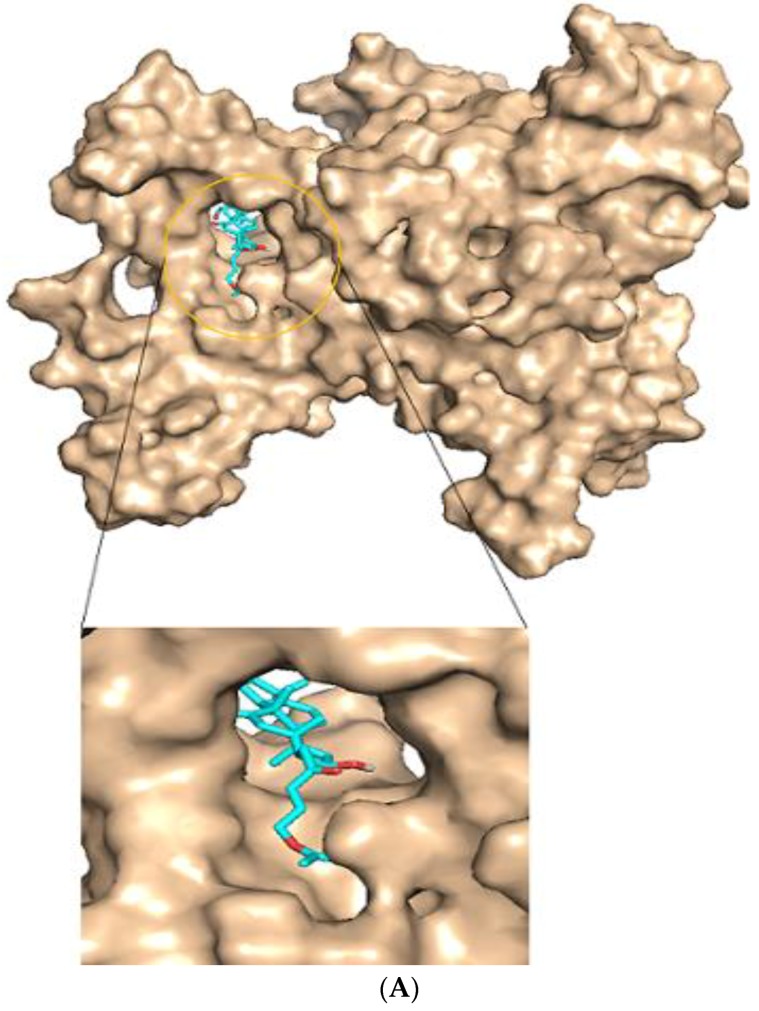

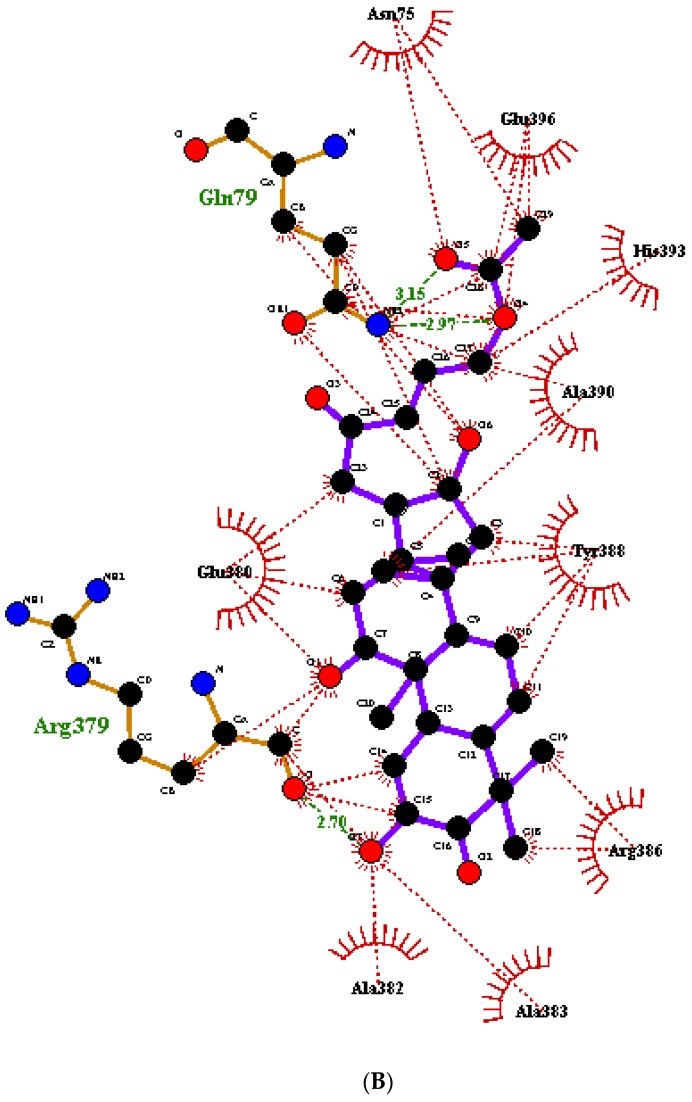
Docking studies and Ligplot+ analysis of Lead molecules. (**A**) Surface representation of a docked complex. ZINC95486305 in sticks (sea-blue color) representation docks firmly within the active site pocket. (**B**) Ligplot diagram of ZINC95486305 lead molecule, purple colored, interacts strongly via three hydrogen bonds with residues Gln79 and Arg379.

Therefore, the proposed lead molecules were ZINC38143792 (Euscaphic acid), ZINC95485880 (hydroxy-(hydroxymethyl)-dimethyl-BLAHone) and ZINC95486305 ([(*E*)-5-[(2*S*,8*S*,9*S*,10*S*,13*S*,14*R*,16*R*,17*S*)-2,16-dihydroxy-4,4,9,13,14-pentamethyl-3,11-dioxo-2,7,8,10,12) (Table 6). ZINC38143792, ZINC95485880, and ZINC95486305 had plausible binding energies (high affinity) of −8.6 kcal/mol, −8.6 kcal/mol, and −8.8 kcal/mol, respectively. ZINC38143792, ZINC95485880, and ZINC95486305 were also observed to have strong hydrogen bond interactions with two or more residues in the active site of the target. ZINC38143792 formed hydrogen bond interactions with three residues (Glu380, Arg379, and Ser357). ZINC95485880 also formed strong hydrogen bond interactions with three residues (Glu380, Arg386, and His393), and lastly, ZINC95486305 also formed hydrogen bond interactions with two Gln79 and Arg379 (Figure 9 and Figure S7). ZINC38143792, ZINC95485880, and ZINC95486305 also had good ADMET properties with lower molecular masses of 487.701 Da, 416.561 Da, and 500.362 Da, respectively. Compounds that have been specifically screened against ICL of *M. tuberculosis* with IC_50_ ≤ 5 µM [70] were obtained from BindingDB [71]. We considered only compounds with IC_50_ ≤ 5 since a compound with 0.06 µM ≤ IC_50_ ≤ 5 µM is considered active, 5 µM ≤ IC50 ≤ 10 µM is considered as weakly active, and IC50 > 10 µM is considered as inactive [32]. The binding energies of the compounds with identifications CHEMBL1818383, CHEMBL1818381, CHEMBL1818380, and CHEMBL1818382 after screening against the model of ICL of *M. ulcerans* are −7.3 kcal/mol, −7.5 kcal/mol, −7.5 kcal/mol, and −7.4 kcal/mol, respectively, which are higher than those of the top 20 (Table 2 and Table S2).

Among the lead molecules, Euscaphic acid was isolated from Hoslundia opposita, which is an aromatic medicinal herb that grows all over Mozambique. Euscaphic acid exhibited a minimum inhibitory concentration of 50 µg mL^−1^ against a drug-sensitive strain of *M. tuberculosis* [72]. Since *Mycobacterium ulcerans* is a close homologue of *Mycobacterium tuberculosis*, Euscaphic acid can be screened against the ICL of *Mycobacterium ulcerans* as a possible inhibitor. Additionally, the biological activities predicted by PASS [73,74] relevant to *M. ulcerans* were dermatological and antibacterial for the other two leads. PASS predicted dermatological and antibacterial activity for ZINC95486305 with probable activity (Pa) and probable inactivity (Pi) values of 0.507 and 0.031 for dermatology, and Pa and Pi values of 0.354 and 0.042 for antibacterial activity, respectively. Similarly, PASS predicted dermatological activity for ZINC95485880 with Pa and Pi values of 0.300 and 0.087, respectively. Pa is based on the probability that a compound under investigation belongs to a subclass of active compounds and Pi is the probability that it belongs to a subclass of inactive compounds within PASS training datasets. When the Pa of a compound is greater than Pi, there is a drive to further investigate the pharmacological activity [74]. Since the Pa values in both dermatological and antibacterial activities were more than the Pi, it is necessary to explore the predicted pharmacological properties of the two leads *in vitro*. To support the aforementioned, a study had shown that herbal preparations with dermatological and antimicrobial properties possessed *anti-M. ulcerans* activity [75].

### 2.10. Induced Fit Docking

**Figure 10:**
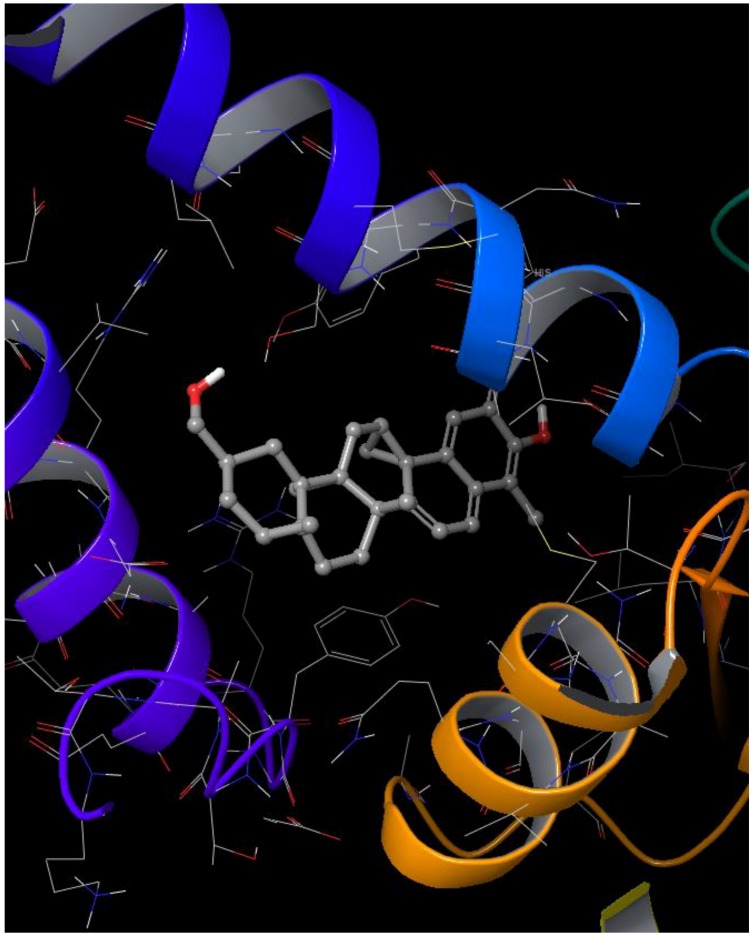
Induced-fit docking studies of ZINC95485880 ligand complex. The Figure illustrates the induced fit pose of ZINC95485880 (shades of gray) docked in the active site of ICL.

**Figure 11:**
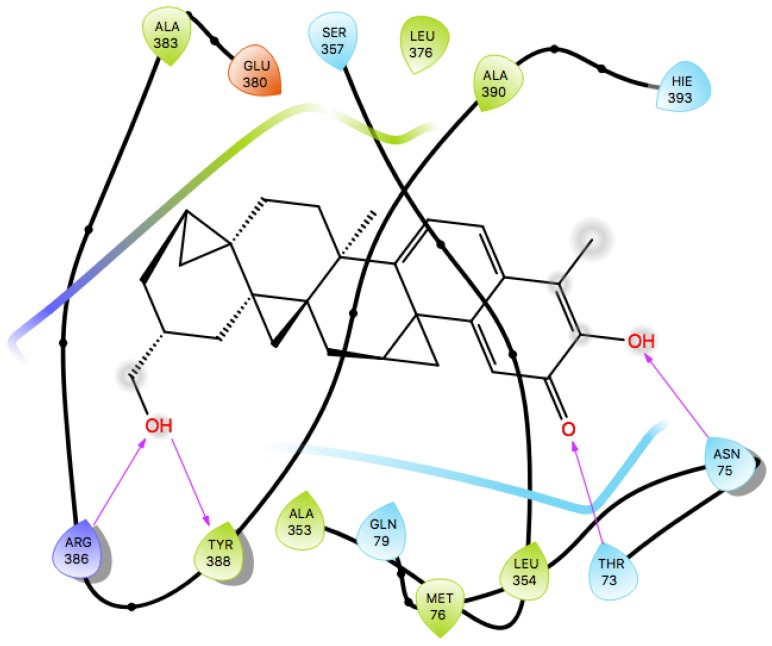
Two-dimensional (2D) representation of molecular interactions of ZINC95485880 ligand complex. Purple arrows represent the hydrogen bonds.

Docking techniques have emerged as useful tools in drug design to virtually screen libraries with the aim of discovering new inhibitors of protein targets. However, the authenticity and credibility of docking predictions are sometimes constrained by difficulties in modeling protein flexibility during ligand binding [76,77]. Induced-fit docking (IFD) was used to validate the predicted leads since IFD consider both the ligands and receptors as flexible [78,79]. The GlideScore, which is used to rank resulting complexes after induced-fit docking, is an empirical scoring function that provides an estimate of the binding affinity between a ligand and a receptor. Lower GlideScores are mostly representative of reasonably good binding between a ligand and a receptor [80,81,82]. Therefore, the more negative the GlideScores, the more plausible the binding [83]. The values obtained for docking scores and GlideScores were the same for all the three lead complexes. The complexes of ZINC95485880, ZINC95486305, and ZINC38143792 had GlideScores of −7.182 kcal/mol, −6.808 kcal/mol, and −5.449 kcal/mol as well as IFD scores of −883.740 kcal/mol, −885.405 kcal/mol, and −892.462 kcal/mol, respectively. The induced-fit pose and molecular interactions of ZINC95485880 are shown in Figure 10 and Figure 11, while those of ZINC95486305 and ZINC38143792 are shown in Figures S8 and S9, respectively. The IFD scores estimate the most plausible conformations of the ligand complex [84]. Lower IFD scores usually represent favorable binding [85] and they are calculated using Equation (1). The three ligand complexes subjected to induced-fit docking formed major interactions with residues comprising THR73, ASN75, GLU380, ARG386, HIS352, ARG379, and SER357, which were also predicted to be key interacting residues within the active site of ICL after molecular interaction analysis with LigPlot+ (Table 2 and Table S2).

Equation (1): IFDScore = 1.0 × Prime_Energy + 9.057 × GlideScore + 1.428 × Glide_Ecoul [86,87]. The Prime_Energy is the total energy of the system while the Glide_Ecoul is the Coulomb term (Coulomb energy).

## 3. Materials and Methods

### 3.1. Sequence Retrieval and Homology Modeling

The protein sequence of isocitrate lyase was retrieved from the National Center for Biotechnology Information (NCBI) database. The sequence was compared to all sequences of available 3D structures stored in the Protein Data Bank using Basic Logical Alignment and Search Tool (BLAST) in order to find suitable templates. Modeller 9.17 [41] was then used to model the 3D structure of the isocitrate lyase using the selected template sequences via the homology modeling process.

### 3.2. Protein Structure Refinement

After homology modeling, all potentially available bumps and clashes in the protein structure were removed using the WHAT IF server [88] by rotating side chain torsion angles and checking all contact distances between atom pairs. ModRefiner was then used to refine the protein model by drawing the model close to its native state in terms of its side-chain positioning, backbone topology, and hydrogen bonds [46]. It was also used to generate significant improvements in the local structure.

### 3.3. Molecular Dynamics Simulation of Protein Structure

The refined structure of the protein model was subjected to molecular dynamics simulation using Gromacs 5.1.1 [47,48,49] with OPLS-AA as a force field. To carry out the simulation, the initial protein structure was solvated in a cubical box using the SPC/E water model, which is a generic equilibrated three-point solvent model. The solvated system contained a charged protein with a net charge of −13 electrons. 13 sodium Na^+^ counter ions were added to neutralize the net charge. The solvated electro-neutral system was then assembled for energy minimization to ensure that the system had no steric clashes or inappropriate geometry. The energy of the relaxed structure was first minimized using the steepest decent method. Equilibration was conducted under two phases comprising NVT and NPT ensembles. The NVT ensemble is conducted under a constant number of particles, volume, and temperature. The pressure was conducted under NPT ensemble with the number of particles, pressure, and temperature kept constant. Both the NVT ensemble and the NPT ensemble were run for 100 ps. The temperature was set to 310 K (37 °C), which represents the normal physiological temperature for the human body. Upon completion of the two equilibration phases, position restraints were released and production molecular dynamics (MD) was run for 1 ns.

### 3.4. Protein Validation and Active Site Prediction

The protein model generated from Modeller 9.17 after its refinement was validated using PROSA [51], PROQ [54], PROCHECK [55], and other applications from the SWISS-MODEL [89,90], including QMEAN.

CASTp [91,92] was used to determine the most plausible binding pockets of the protein model. The computation of the binding site is based on quantitative characterization of surface pockets and internal voids, which are the important concave regions associated with binding properties of the protein structure [91]. The predicted cavity by CASTp was confirmed by the blind docking [93] process using AutoDock Vina within PyRx software version 0.8 [94,95].

### 3.5. Molecular Docking and Mechanisms of Binding

A library of 885 natural compounds were retrieved from AfroDb for docking against the protein model. AfroDb [32] is a database that contains African natural compounds and a subset catalogue of the ZINC database [96]. All the AfroDb drug-like compounds were retrieved in an SDF format in a single file. The ligands were energy minimized using Open Babel in PyRx (Version 0.8) prior to docking in order to obtain 3D ligand structures with proper bond lengths between atoms. Energy minimization was carried using the Universal Force Field (UFF), which is reasonably good for molecules containing elements found within the periodic Table [97]. The optimization algorithm employed in this study was the conjugate algorithm. This was followed by conversion of all the ligands into AutoDock PDBQT format. AutoDock Vina embedded in the PyRx software was used to perform molecular docking and virtual screening keeping the ligands flexible and the receptor rigid. Each ligand was allowed nine conformers for every docking process. Known inhibitors and ligands obtained from BindingDB [71,98], PDB [36,99], and literature were screened against the generated homology model of ICL of *Mycobacterium ulcerans* and the crystal structures of *M. tuberculosis*. The mechanisms of binding between the ligand-receptor complexes were profiled using Ligplot+ [100]. For most docking programs, a flexible ligand docks to rigid receptor but understanding the flexibility of the receptor is very vital to conformational changes since most protein structures experience side-chain or backbone movement upon binding to a ligand [77,83]. Induced Fit Docking (IFD) of the leads was done using the IFD module in the Schrödinger software suite [79]. The GlideScore and IFD score were generated for each pose.

### 3.6. Validation of Docking Protocol

In order to validate the docking protocol, ligands with experimentally determined pose within crystallographic protein structures were extracted from receptor-ligand complexes and re-docked to the receptors [101]. The complexes used were templates of the ICL of *M. tuberculosis*, a close homologue of *M. ulcerans* [16,102] and they were 5DQL, 1F8I, and 1F8M with their co-crystallized ligands comprising 4-hydroxy-2-oxobutanoic acid, succinic acid, glyoxylic acid, and pyruvic acid. The co-crystalized ligands were initially removed from their respective protein active sites and later re-docked into the receptors, which was done previously [60]. The predicted docking poses were compared to their corresponding crystallographic complexes and were superimposed in order to assess how well they align to each other and their common residues of molecular interactions. These four ligands were also docked within the modeled structure of ICL of *M. ulcerans*. Additionally, LigAlign was used to align the re-docked ligand complexes with the ligands in the co-crystalized complexes. LigAlign enables the analysis of ligand alignments within active sites [59].

As part of the validation of the molecular docking, the four known ligands in the co-crystalized structures of *M. tuberculosis* were extracted from the structures of the complexes comprising 1F8I, 1F8M, and 5DQL to aid in generating the receiver operating characteristics (ROC) curve. The ligands, which include succinic acid, glyoxylic acid, pyruvic acid, and 4-hydroxy-2-oxobutanoic acid, were used to obtain decoys via the Directory of useful decoys and enhanced (DUD-E) [103]. The decoys generated have similar physicochemical properties to the ligands but different 2-D topology. The number of generated decoys of succinic acid, glyoxylic acid, pyruvic acid, and 4-hydroxy-2-oxobutanoic acid were 50, 50, 50, and 49, respectively. Duplicated protonated ligands and their corresponding decoys were eliminated to prevent analog bias [62]. A total of 199 decoys and the four active ligands were screened separately against the ICL model structure of *M. ulcerans*, 1F8I, 1F8M, and 5DQL in order to calculate the area under the curve (AUC) value of the computed ROC curve. The ROC curves were generated with default settings using easyROC (Ver. 1.3), which operates in the R language environment [63]. The default settings consist of non-parametrically fitted ROC curve, type I error of 0.05, Standard error estimation of DeLong (1988), and confidence interval of DeLong (1988) [104].

### 3.7. Pharmacological Profiling

The top hits were pharmacologically profiled via Absorption, Distribution, Metabolism, Excretion, and Toxicity (ADME/Tox) testing using the ADMET Predictor 8.0 developed by SimulationsPlus and SwissADME [69].

### 3.8. Prediction of Activity Spectra for Substances (PASS) for Leads

The SMILES files of ZINC95486305 and ZINC95485880 were used to predict the biological activity using the prediction of activity spectra for substances (PASS) tool [73]. PASS is a tool that predicts over 3500 different kinds of biological activity, pharmacological effects, and mechanisms of action, toxicity, and interactions with metabolic enzymes and transporters as well as influence gene expression. The PASS algorithm is based on the analysis of structure activity relationships for over 250,000 bioactive substances including drugs, drug candidates, leads, and toxic compounds. PASS, therefore, estimates the probability that a compound belongs to a particular class of active compounds.

## 4. Conclusions

Isocitrate lyase is a key enzyme in the glyoxylate cycle of *Mycobacterium ulcerans*, and in this present study, its 3D structure was successfully generated using homology modeling techniques. In addition, molecular dynamics simulation was successfully performed on the predicted protein model. Furthermore, using molecular docking, 885 natural compounds retrieved from AfroDb were screened via the predicted active site of the modeled structure. Out of 885 compounds, 20 hit compounds were found based on a low binding energy (strong binding affinity) and reasonable docking pose upon visualization. An ROC curve with a reasonably good AUC value of 0.89375 was used to validate the docking protocol for the model structure of ICL of *M. ulcerans*. The hit compounds were further analyzed using ADMET testing and physicochemical profiling. Therefore, we propose that ZINC38143792, ZINC95485880, and ZINC95486305 are the lead compounds since they emerged as the best compounds among the 20 hits based on their favorable ADMET properties and strong active site interactions. ZINC38143792, which is also known as Euscaphic acid, has been reported to inhibit a drug-sensitive strain of *M. tuberculosis* [72]. ZINC95486305 and ZINC95485880 were both predicted to possess dermatological activity while ZINC95486305 also had antibacterial properties. If the efficiencies of the leads are successfully proven via biochemical assays, these molecules could be important inhibitors to ICL, which is an essential target in the Buruli ulcer disease mechanism. The predicted leads can serve as scaffolds for further development of potent anti-buruli ulcer drugs.

## Figures and Tables

**Table 1:** A Table showing five successfully generated models with Modeller 9v17. The DOPE score is mainly based on probability theory and it provides information on the energy of the protein generated via Modeller 9v17.

Models	DOPE Score
Model 1	−47200.15625
Model 2	−47099.78906
Model 3	−47185.48047
Model 4	−47193.96484
Model 5	−47291.21875

**Table 2:** A Table showing 10 of the selected ligands. The binding energies of the ligands are shown in kcal/mol. The residues within the active site of the ICL protein that are interacting with the ligands via hydrogen bonding and hydrophobic interactions are shown.

Predicted Ligands	Binding Energy/(Kcal/mol)	Hydrogen Bond Interacting Residues	Hydrophobic Bond Interacting Residues
ZINC95486006	−9.5	Asn75, Ser357, Glu380, Ala390	Met76, Gln79, Ala353, Leu354, Met358, Leu361, Ala362, Tyr365, Tyr373, Leu376, His393, Glu396
ZINC95486007	−8.7	Glu380, Asn75, Ala390	Met76, Gln79, Leu354, Ser357, Met358, Leu361, Ala362, Tyr365, Tyr373, Leu376, His393, Glu396
ZINC38143792	−8.6	Glu380, Arg379, Ser357	Gln79, Ala382, Ala383, Arg386, Tyr388, Ala390
ZINC95485880	−8.6	Glu380, Arg386, His393	Asn75, Met76, Gln79, Ala383, Tyr388
ZINC95486305	−8.8	Gln79, Arg379	Asn75, Glu396, His393, Ala390, Glu380, Tyr388, Arg386, Ala382, Ala383
ZINC95486303	−8.7	Asn319, Lys321	Asn67, Leu69, Gln79, Gln80, Ala83, Leu85, Pro316, Trp320, Ile329, Ile346, Ala349, Ala353, Tyr388
ZINC95485905	−8.5	Glu380, His393	Asn75, Gln79, Leu376, Arg379, Ala383, Tyr388, Ala390
ZINC95486183	−10.0	Glu380	Leu69, Met76, Asn75, Gln79, Pro316, Trp320, Ile346, Ala349, Ala353, Leu376, Trp388, Ala390, His393, Glu396, Val397
ZINC95486184	−9.6	Ala349	Leu69, Asn75, Met76, Gln79, Pro316, Trp320, Ile346, Gly350, His352, Ala353, Leu354, Ser357, Tyr388
ZINC95486142	−9.4	Pro316	Ser315, Ser317, Asn319, Trp320, Lys321, Ile346, Ala349, His352, Asn355

**Table 3:** Drug-likeness and water solubility of the top five (5) hits and five (5) known drugs (where No. HA = Number of H-bond acceptors, MW = Molecular weight, No. HD = Number of H-bond donors, Bio Sc = Bioavailability Score).

Compound ZINC ID/Name	Number of Lipinski’s Rules Violated	MW (g/mol)	No. HA	No. HD	xLogP	Water Solubility (mg/mL)	Log S	Bio. Sc
ZINC95486006	3	666.805	12	7	0.86	Moderately soluble	−4.46	0.17
ZINC95486007	3	668.821	12	7	1.02	Moderately soluble	−4.86	0.17
ZINC38143792	0	487.701	5	3	4.93	Moderately soluble	−5.92	0.56
ZINC95485880	0	416.561	3	2	3.79	Moderately soluble	−5.03	0.55
ZINC95486305	1	500.362	7	2	2.49	Soluble	−3.72	0.55
RIFAMPICIN	3	822.94	14	6	3.07	Poorly soluble	−8.18	0.17
STREPTOMYCIN	3	581.57	15	11	−5.83	Soluble	1.80	0.17
CLARITHROMYCIN	2	747.95	14	4	2.13	Moderately soluble	−5.94	0.17
MOXIFLOXACIN	0	401.43	6	3	1.85	Soluble	−2.70	0.55
AMIKACIN	3	585.60	17	13	−5.91	Highly Soluble	2.23	0.17

**Table 4:** Pharmacokinetics properties of predicted compounds and five known drugs. The pharmacokinetics properties comprised cytochrome inhibition, the blood brain barrier permeant (BBB), P-glycoprotein (P-gp) substrates, and gastrointestinal (GI) absorption.

Compound ZINC ID	GI Absorption	BBB Permeant	P-gp Substrate	CYP1A2 Inhibitor	CYP2C19 Inhibitor	CYP2C9 Inhibitor	CYP2D6 Inhibitor	CYP3A4 Inhibitor
ZINC95486006	Low	No	Yes	No	No	No	No	No
ZINC95486007	Low	No	Yes	No	No	No	No	No
ZINC38143792	High	No	Yes	No	No	No	No	No
ZINC95485880	High	Yes	Yes	No	No	No	No	No
ZINC95486305	High	No	Yes	No	No	No	No	Yes
ZINC95486303	Low	No	Yes	No	No	No	No	Yes
ZINC95485905	Low	No	No	No	No	Yes	No	No
ZINC95486183	Low	No	Yes	No	No	No	No	No
ZINC95486184	Low	No	Yes	No	No	No	No	No
ZINC95486142	Low	No	No	No	No	No	No	No
ZINC86037206	High	No	No	No	No	Yes	No	Yes
ZINC31761332	Low	No	No	No	No	Yes	No	Yes
ZINC95486231	High	No	Yes	No	No	No	No	No
ZINC03197457	Low	No	No	No	No	No	No	No
ZINC95485943	High	No	Yes	No	No	No	No	No
ZINC95486001	High	No	Yes	No	No	Yes	No	No
ZINC40431237	High	No	No	No	No	Yes	No	Yes
ZINC95486182	Low	No	No	No	No	No	No	No
ZINC03941105	High	No	No	No	No	Yes	No	No
ZINC95485882	Low	No	No	No	No	No	No	No
RIFAMPICIN	Low	No	Yes	No	No	No	No	No
STREPTOMYCIN	Low	No	Yes	No	No	No	No	No
CLARITHROMYCIN	Low	No	Yes	No	No	No	No	No
MOXIFLOXACIN	High	No	Yes	No	No	No	Yes	No
AMIKACIN	Low	No	Yes	No	No	No	No	No

**Table 5:** Cardiac Toxicity and Mutagenicity tests. Cardiac toxicity was based on the hERG model, which predicts whether the compound blocks the hERG K+ channel or not. A “yes” indicate a compound that has the likelihood to block a channel and a “no” indicate otherwise and “negative” means compound might not cause any mutation in host genes.

Compounds ZINC ID	Cardiac Toxicity	Mutagenicity
ZINC95486006	No	Negative
ZINC95486007	No	Negative
ZINC38143792	No	Negative
ZINC95485880	No	Negative
ZINC95486305	No	Negative
ZINC95486303	No	Negative
ZINC95485905	No	Negative
ZINC95486183	No	Negative
ZINC95486184	No	Negative
ZINC95486142	Yes	Negative
ZINC86037206	No	Negative
ZINC31761332	No	Negative
ZINC95486231	No	Negative
ZINC03197457	No	Negative
ZINC95485943	No	Negative
ZINC95486001	No	Negative
ZINC40431237	No	Negative
ZINC95486182	Yes	Negative
ZINC03941105	No	Negative
ZINC95485882	No	Negative

**Table 6:** A table showing the two-dimensional (2D) structures of three (3) selected leads generated with DrugBank (https://www.drugbank.ca/).

ZINC38143792	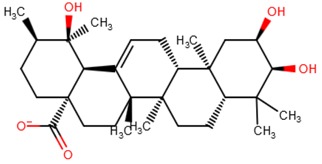
ZINC95485880	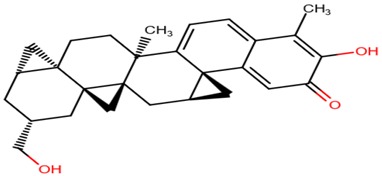
ZINC95486305	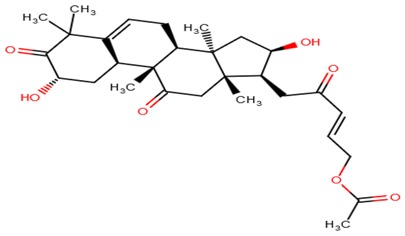

## Supplementary Materials

Figures S1–S9, and Tables S1–S3 are available as supplementary materials accessible online.

## References

[1] Oliveira M.S., Fraga A.G., Torrado E., Castro A.G., Pereira J.P., Filho A.L., Milanezi F., Schmitt F.C., Meyers W.M., Portaels F. (2005). Infection with *Mycobacterium ulcerans* Induces Persistent Inflammatory Responses in Mice. Infect. Immun..

[2] Wilson M.D., Boakye D.A., Mosi L., Asiedu K. (2011). In the case of transmission of *Mycobacterium ulcerans* in buruli ulcer disease *Acanthamoeba* species stand accused. Ghana Med. J..

[3] Leão S.C., Romano M.I., Jesus M. (2007). Tuberculosis, Leprosy, and Other Mycobacterioses. Bioinformatics in Tropical Disease Research: A Practical and Case-Study Approach.

[4] Marsollier L., Robert R., Aubry J., Saint André J.-P., Kouakou H., Legras P., Manceau A.-L., Mahaza C., Carbonnelle B. (2002). Aquatic insects as a vector for *Mycobacterium ulcerans*. Appl. Environ. Microbiol..

[5] Marsollier L., Deniaux E., Brodin P., Marot A., Wondje C.M., Saint-André J.-P., Chauty A., Johnson C., Tekaia F., Yeramian E. (2007). Protection against *Mycobacterium ulcerans* Lesion Development by Exposure to Aquatic Insect Saliva. PLoS Med..

[6] Merritt R.W., Walker E.D., Small P.L.C., Wallace J.R., Johnson P.D.R., Benbow M.E., Boakye D.A. (2010). Ecology and transmission of Buruli ulcer disease: A systematic review. PLoS Negl. Trop. Dis..

[7] World Health Organization (WHO) (2012). Treatment of Mycobacterium Ulcerans Disease (Buruli Ulcer).

[8] Azumah B.K., Addo P.G., Dodoo A., Awandare G., Mosi L., Boakye D.A., Wilson M.D. (2017). Experimental demonstration of the possible role of *Acanthamoeba polyphaga* in the infection and disease progression in Buruli Ulcer (BU) using ICR mice. PLoS ONE.

[9] WHO (2017). Buruli Ulcer.

[10] Kumar S., Basu S., Bhartiya S.K., Shukla V.K. (2015). The Buruli Ulcer. Int. J. Low. Extrem. Wounds.

[11] Klis S., Stienstra Y., Phillips R.O., Abass K.M., Tuah W., van der Werf T.S. (2014). Long Term Streptomycin Toxicity in the Treatment of Buruli Ulcer: Follow-up of Participants in the BURULICO Drug Trial. PLoS Negl. Trop. Dis..

[12] Yeboah-Manu D., Kpeli G.S., Ruf M.-T., Asan-Ampah K., Quenin-Fosu K., Owusu-Mireku E., Paintsil A., Lamptey I., Anku B., Kwakye-Maclean C. (2013). Secondary bacterial infections of buruli ulcer lesions before and after chemotherapy with streptomycin and rifampicin. PLoS Negl. Trop. Dis..

[13] Gordon C.L., Buntine J.A., Hayman J.A., Lavender C.J., Fyfe J.A.M., Hosking P., Starr M., Johnson P.D.R. (2010). All-Oral Antibiotic Treatment for Buruli Ulcer: A Report of Four Patients. PLoS Negl. Trop. Dis..

[14] Ji B., Lefrancois S., Robert J., Chauffour A., Truffot C., Jarlier V. (2006). In Vitro and In Vivo Activities of Rifampin, Streptomycin, Amikacin, Moxifloxacin, R207910, Linezolid, and PA-824 against *Mycobacterium ulcerans*. Antimicrob. Agents Chemother..

[15] Ji B., Chauffour A., Robert J., Lefrançois S., Jarlier V. (2007). Orally administered combined regimens for treatment of *Mycobacterium ulcerans* infection in mice. Antimicrob. Agents Chemother..

[16] Scherr N., Pluschke G., Panda M. (2016). Comparative Study of Activities of a Diverse Set of Antimycobacterial Agents against *Mycobacterium tuberculosis* and *Mycobacterium ulcerans*. Antimicrob. Agents Chemother..

[17] Tsouh P.V.F., Addo P., Yeboah-Manu D., Boyom F.F. (2015). Methods used in preclinical assessment of anti-Buruli ulcer agents: A global perspective. J. Pharmacol. Toxicol. Methods.

[18] Buruli Ulcer (*Mycobacterium ulcerans* Infection). http://www.who.int/en/news-room/fact-sheets/detail/buruli-ulcer-(mycobacterium-ulcerans-infection.

[19] Johnson P.D.R., Stinear T., Small P.L.C., Pluschke G., Merritt R.W., Portaels F., Huygen K., Hayman J.A., Asiedu K. (2005). Buruli Ulcer (*M. ulcerans* Infection): New Insights, New Hope for Disease Control. PLoS Med..

[20] Katiyar C., Gupta A., Kanjilal S., Katiyar S. (2012). Drug discovery from plant sources: An integrated approach. Ayu.

[21] Talele T., Khedkar S., Rigby A. (2010). Successful Applications of Computer Aided Drug Discovery: Moving Drugs from Concept to the Clinic. Curr. Top. Med. Chem..

[22] Zhang G., Guo S., Cui H., Qi J. (2018). Virtual Screening of Small Molecular Inhibitors against DprE1. Molecules.

[23] Billones J., Carrillo M., Organo V., Macalino S.J., Emnacen I., Bernadette J. (2013). Virtual Screening against *Mycobacterium tuberculosis* Lipoate Protein Ligase B (MtbLipB) and In Silico ADMET Evaluation of Top Hits. Orient. J. Chem..

[24] Kumar A., Siddiqi M.I. (2008). Virtual screening against *Mycobacterium tuberculosis* dihydrofolate reductase: Suggested workflow for compound prioritization using structure interaction fingerprints. J. Mol. Graph. Model..

[25] McKinney J.D., zu Bentrup K.H., Muñoz-Elías E.J., Miczak A., Chen B., Chan W.-T., Swenson D., Sacchettini J.C., Jacobs W.R., Russell D.G. (2000). Persistence of *Mycobacterium tuberculosis* in macrophages and mice requires the glyoxylate shunt enzyme isocitrate lyase. Nature.

[26] Dunn M.F., Ramirez-Trujillo J.A., Hernandez-Lucas I. (2009). Major roles of isocitrate lyase and malate synthase in bacterial and fungal pathogenesis. Microbiology.

[27] Lee Y.-V., Wahab H.A., Choong Y.S. (2015). Potential inhibitors for isocitrate lyase of *Mycobacterium tuberculosis* and non-*M. tuberculosis*: A summary. Biomed. Res. Int..

[28] Kim D., Lee J., Lee S., Park J., Lee D. (2016). Predicting unintended effects of drugs based on off-target tissue effects. Biochem. Biophys. Res. Commun..

[29] Chartier M., Morency L.-P., Zylber M.I., Najmanovich R.J. (2017). Large-scale detection of drug off-targets: Hypotheses for drug repurposing and understanding side-effects. BMC Pharmacol. Toxicol..

[30] Chang R.L., Xie L., Xie L., Bourne P.E., Palsson B.Ø. (2010). Drug off-target effects predicted using structural analysis in the context of a metabolic network model. PLoS Comput. Biol..

[31] Butt A.M., Nasrullah I., Tahir S., Tong Y. (2012). Comparative Genomics Analysis of *Mycobacterium ulcerans* for the Identification of Putative Essential Genes and Therapeutic Candidates. PLoS ONE.

[32] Ntie-Kang F., Zofou D., Babiaka S.B., Meudom R., Scharfe M., Lifongo L.L., Mbah J.A., Mbaze L.M., Sippl W., Efange S.M.N. (2013). AfroDb: A Select Highly Potent and Diverse Natural Product Library from African Medicinal Plants. PLoS ONE.

[33] Alvin A., Miller K.I., Neilan B.A. (2014). Exploring the potential of endophytes from medicinal plants as sources of antimycobacterial compounds. Microbiol. Res..

[34] Dankwa B., Kwofie K.S. In Silico Prediction of Potential Natural Product-Derived Lead Compounds for the Treatment of Buruli Ulcer. Proceedings of the Waccbip Research Conference.

[35] Benson D.A., Clark K., Karsch-Mizrachi I., Lipman D.J., Ostell J., Sayers E.W. (2015). GenBank. Nucleic Acids Res..

[36] Rose P.W., Prlić A., Altunkaya A., Bi C., Bradley A.R., Christie C.H., Di Costanzo L., Duarte J.M., Dutta S., Feng Z. (2017). The RCSB protein data bank: Integrative view of protein, gene and 3D structural information. Nucleic Acids Res..

[37] Rose P.W., Prlić A., Bi C., Bluhm W.F., Christie C.H., Dutta S., Green R.K., Goodsell D.S., Westbrook J.D., Woo J. (2015). The RCSB Protein Data Bank: Views of structural biology for basic and applied research and education. Nucleic Acids Res..

[38] Johnson M., Zaretskaya I., Raytselis Y., Merezhuk Y., McGinnis S., Madden T.L. (2008). NCBI BLAST: A better web interface. Nucleic Acids Res..

[39] Altschul S.F., Gish W., Miller W., Myers E.W., Lipman D.J. (1990). Basic local alignment search tool. J. Mol. Biol..

[40] Biasini M., Bienert S., Waterhouse A., Arnold K., Studer G., Schmidt T., Kiefer F., Cassarino T.G., Bertoni M., Bordoli L. (2014). SWISS-MODEL: Modelling protein tertiary and quaternary structure using evolutionary information. Nucleic Acids Res..

[41] Eswar N., Webb B., Marti-Renom M.A., Madhusudhan M.S., Eramian D., Shen M., Pieper U., Sali A. (2007). Comparative Protein Structure Modeling Using MODELLER. Curr. Protoc. Protein Sci..

[42] Shen M.-Y., Sali A. (2006). Statistical potential for assessment and prediction of protein structures. Protein Sci..

[43] Webb B., Sali A. (2016). Comparative protein structure modeling using MODELLER. Current Protocols in Bioinformatics.

[44] Fiser A. (2010). Template-Based Protein Structure Modeling. Methods in Molecular Biology.

[45] Hasan M.A., Alauddin S.M., Al Amin M., Nur S.M., Mannan A. (2014). In silico molecular characterization of cysteine protease YopT from Yersinia pestis by homology modeling and binding site identification. Drug Target Insights.

[46] Xu D., Zhang Y. (2011). Improving the Physical Realism and Structural Accuracy of Protein Models by a Two-Step Atomic-Level Energy Minimization. Biophys. J..

[47] Abraham M.J., Murtola T., Schulz R., Páll S., Smith J.C., Hess B., Lindahl E. (2015). GROMACS: High performance molecular simulations through multi-level parallelism from laptops to supercomputers. SoftwareX.

[48] Bekker H., Berendsen H., Dijkstra E., Achterop S., Vondrumen R., Vanderspoel D., Sijbers A., Keegstra H., Renardus M. (1993). Gromacs—A Parallel Computer for Molecular-Dynamics Simulations.

[49] Van Der Spoel D., Lindahl E., Hess B., Groenhof G., Mark A.E., Berendsen H.J.C. (2005). GROMACS: Fast, flexible, and free. J. Comput. Chem..

[50] Fiser A., Sali A. (2003). ModLoop: Automated modeling of loops in protein structures. Bioinformatics.

[51] Wiederstein M., Sippl M.J. (2007). ProSA-web: Interactive web service for the recognition of errors in three-dimensional structures of proteins. Nucleic Acids Res..

[52] Yakubu A., De Donato M., Imumorin I.G. (2017). Modelling functional and structural impact of non-synonymous single nucleotide polymorphisms of the DQA1 gene of three Nigerian goat breeds. S. Afr. J. Anim. Sci..

[53] Cristobal S., Zemla A., Fischer D., Rychlewski L., Elofsson A. (2001). A study of quality measures for protein threading models. BMC Bioinform..

[54] Wallner B., Elofsson A. (2003). Can correct protein models be identified?. Protein Sci..

[55] Laskowski R.A., MacArthur M.W., Moss D.S., Thornton J.M. (1993). PROCHECK: A program to check the stereochemical quality of protein structures. J. Appl. Crystallogr..

[56] Meng X.-Y., Zhang H.-X., Mezei M., Cui M. (2011). Molecular docking: A powerful approach for structure-based drug discovery. Curr. Comput. Aided. Drug Des..

[57] Patil R., Das S., Stanley A., Yadav L., Sudhakar A., Varma A.K. (2010). Optimized Hydrophobic Interactions and Hydrogen Bonding at the Target-Ligand Interface Leads the Pathways of Drug-Designing. PLoS ONE.

[58] Pereira A.L.E., dos Santos G.B., Franco M.S.F., Federico L.B., da Silva C.H.T.P., Santos C.B.R. (2018). Molecular modeling and statistical analysis in the design of derivatives of human dipeptidyl peptidase IV. J. Biomol. Struct. Dyn..

[59] Heifets A., Lilien R.H. (2010). LigAlign: Flexible ligand-based active site alignment and analysis. J. Mol. Graph. Model..

[60] Alves M.J., Froufe H.J.C., Costa A.F.T., Santos A.F., Oliveira L.G., Osório S.R.M., Abreu R.M.V., Pintado M., Ferreira I.C.F.R. (2014). Docking studies in target proteins involved in antibacterial action mechanisms: Extending the knowledge on standard antibiotics to antimicrobial mushroom compounds. Molecules.

[61] Triballeau N., Acher F., Brabet I., Pin J.-P., Bertrand H.-O. (2005). Virtual screening workflow development guided by the “receiver operating characteristic” curve approach. Application to high-throughput docking on metabotropic glutamate receptor subtype 4. J. Med. Chem..

[62] Shamsara J. (2018). Correlation between Virtual Screening Performance and Binding Site Descriptors of Protein Targets. Int. J. Med. Chem..

[63] Goksuluk D., Korkmaz S., Zararsiz G., Karaagaoglu A.E. (2016). EasyROC: An interactive web-tool for ROC curve analysis using R language environment. R J..

[64] Cruz J.V., Neto M.F.A., Silva L.B., da Ramos R., da Costa J., Brasil D.S.B., Lobato C.C., da Costa G.V., Bittencourt J.A.H.M., da Silva C.H.T.P. (2018). Identification of Novel Protein Kinase Receptor Type 2 Inhibitors Using Pharmacophore and Structure-Based Virtual Screening. Molecules.

[65] Mandrekar J.N. (2010). Receiver operating characteristic curve in diagnostic test assessment. J. Thorac. Oncol..

[66] El Khouli R.H., Macura K.J., Barker P.B., Habba M.R., Jacobs M.A., Bluemke D.A. (2009). Relationship of temporal resolution to diagnostic performance for dynamic contrast enhanced MRI of the breast. J. Magn. Reson. Imaging.

[67] Lipinski C.A., Lombardo F., Dominy B.W., Feeney P.J. (2001). Experimental and computational approaches to estimate solubility and permeability in drug discovery and development settings. Adv. Drug Deliv. Rev..

[68] Lipinski C.A. (2000). Drug-like properties and the causes of poor solubility and poor permeability. J. Pharmacol. Toxicol. Methods.

[69] Daina A., Michielin O., Zoete V. (2017). SwissADME: A free web tool to evaluate pharmacokinetics, drug-likeness and medicinal chemistry friendliness of small molecules. Sci. Rep..

[70] Sriram D., Yogeeswari P., Methuku S., Vyas D.R.K., Senthilkumar P., Alvala M., Jeankumar V.U. (2011). Synthesis of various 3-nitropropionamides as *Mycobacterium tuberculosis* isocitrate lyase inhibitor. Bioorg. Med. Chem. Lett..

[71] Gilson M.K., Liu T., Baitaluk M., Nicola G., Hwang L., Chong J. (2016). BindingDB in 2015: A public database for medicinal chemistry, computational chemistry and systems pharmacology. Nucleic Acids Res..

[72] Mujovo S.F., Hussein A.A., Meyer J.J.M., Fourie B., Muthivhi T., Lall N. (2008). Bioactive compounds from *Lippia javanica* and *Hoslundia opposita*. Nat. Prod. Res..

[73] Lagunin A., Stepanchikova A., Filimonov D., Poroikov V. (2000). PASS: Prediction of activity spectra for biologically active substances. Bioinformatics.

[74] Jamkhande P.G., Pathan S.K., Wadher S.J. (2016). In silico PASS analysis and determination of antimycobacterial, antifungal, and antioxidant efficacies of maslinic acid in an extract rich in pentacyclic triterpenoids. Int. J. Mycobacteriol..

[75] Addo P., Quartey M., Abbas M., Adu-Addai B., Owusu E., Okang I., Dodoo A., De Souza D., Ankrah N., Ofori-Adjei D. (2007). In-Vitro Susceptibility of Mycobacterium Ulcerans to Herbal Preparations. Internet J. Trop. Med..

[76] Xu M., Lill M.A. (2013). Induced fit docking, and the use of QM/MM methods in docking. Drug Discov. Today Technol..

[77] Carlson H.A. (2002). Protein flexibility and drug design: How to hit a moving target. Curr. Opin. Chem. Biol..

[78] Farid R., Day T., Friesner R.A., Pearlstein R.A. (2006). New insights about HERG blockade obtained from protein modeling, potential energy mapping, and docking studies. Bioorg. Med. Chem..

[79] Sherman W., Day T., Jacobson M.P., Friesner R.A., Farid R. (2006). Novel procedure for modeling ligand/receptor induced fit effects. J. Med. Chem..

[80] Friesner R.A., Banks J.L., Murphy R.B., Halgren T.A., Klicic J.J., Mainz D.T., Repasky M.P., Knoll E.H., Shelley M., Perry J.K. (2004). Glide: A New Approach for Rapid, Accurate Docking and Scoring. 1. Method and Assessment of Docking Accuracy. J. Med. Chem..

[81] Friesner R.A., Murphy R.B., Repasky M.P., Frye L.L., Greenwood J.R., Halgren T.A., Sanschagrin P.C., Mainz D.T. (2006). Extra Precision Glide: Docking and Scoring Incorporating a Model of Hydrophobic Enclosure for Protein-Ligand Complexes. J. Med. Chem..

[82] Kumar Deokar H., Barch H.P., Buolamwini J.K. (2017). Homology Modeling of Human Concentrative Nucleoside Transporters (hCNTs) and Validation by Virtual Screening and Experimental Testing to Identify Novel hCNT1 Inhibitors. Drug Des..

[83] Zhong H., Tran L.M., Stang J.L. (2009). Induced-fit docking studies of the active and inactive states of protein tyrosine kinases. J. Mol. Graph. Model..

[84] Medina-Franco J.L., Méndez-Lucio O., Yoo J. (2014). Rationalization of activity cliffs of a sulfonamide inhibitor of DNA methyltransferases with induced-fit docking. Int. J. Mol. Sci..

[85] Luo H.J., Wang J.Z., Huang N.Y., Deng W.Q., Zou K. (2014). Induced-fit docking and virtual screening for 8-hydroxy-3-methoxy- 5H-pyrido [2,1-c] pyrazin-5-one derivatives as inducible nitric oxide synthase inhibitors. J. Chem. Pharm. Res..

[86] Sherman W., Beard H.S., Farid R. (2006). Use of an Induced Fit Receptor Structure in Virtual Screening. Chem. Biol. Drug Des..

[87] How Is the IFD Score Calculated and What Is Its Units?. https://www.schrodinger.com/kb/307.

[88] Vriend G. (1990). WHAT IF: A molecular modeling and drug design program. J. Mol. Graph..

[89] Arnold K., Bordoli L., Kopp J., Schwede T. (2006). The SWISS-MODEL workspace: A web-based environment for protein structure homology modelling. Bioinformatics.

[90] Guex N., Peitsch M.C. (1997). SWISS-MODEL and the Swiss-Pdb Viewer: An environment for comparative protein modeling. Electrophoresis.

[91] Binkowski T.A., Naghibzadeh S., Liang J. (2003). CASTp: Computed Atlas of Surface Topography of proteins. Nucleic Acids Res..

[92] Dundas J., Ouyang Z., Tseng J., Binkowski A., Turpaz Y., Liang J. (2006). CASTp: Computed atlas of surface topography of proteins with structural and topographical mapping of functionally annotated residues. Nucleic Acids Res..

[93] Paul B.K., Ray D., Guchhait N. (2013). Unraveling the binding interaction and kinetics of a prospective anti-HIV drug with a model transport protein: Results and challenges. Phys. Chem. Chem. Phys..

[94] Padilha E.C., Serafim R.B., Sarmiento D.Y.R., Santos C.F., Santos C.B.R., Silva C.H.T.P. (2016). New PPARα/γ/δ Optimal Activator Rationally Designed by Computational Methods. J. Braz. Chem. Soc..

[95] Khan M.F., Nahar N., Bin Rashid R., Chowdhury A., Rashid M.A. (2018). Computational investigations of physicochemical, pharmacokinetic, toxicological properties and molecular docking of betulinic acid, a constituent of Corypha taliera (Roxb.) with Phospholipase A2 (PLA2). BMC Complement. Altern. Med..

[96] Irwin J.J., Sterling T., Mysinger M.M., Bolstad E.S., Coleman R.G. (2012). ZINC: A free tool to discover chemistry for biology. J. Chem. Inf. Model..

[97] O’Boyle N.M., Banck M., James C.A., Morley C., Vandermeersch T., Hutchison G.R. (2011). Open Babel: An Open chemical toolbox. J. Cheminform..

[98] Wassermann A.M., Bajorath J. (2011). BindingDB and ChEMBL: Online compound databases for drug discovery. Expert Opin. Drug Discov..

[99] Berman H.M., Westbrook J., Feng Z., Gilliland G., Bhat T.N., Weissig H., Shindyalov I.N., Bourne P.E. (2000). The Protein Data Bank. Nucleic Acids Res..

[100] Laskowski R.A., Swindells M.B. (2011). LigPlot+: Multiple ligand-protein interaction diagrams for drug discovery. J. Chem. Inf. Model..

[101] Da Silva Costa J., da Silva Lopes Costa K., Cruz J.V., da Silva Ramos R., Silva L.B., Do Socorro Barros Brasil D., de Paula da Silva C.H.T., Dos Santos C.B.R., da Cruz Macedo W.J. (2018). Virtual Screening and Statistical Analysis in the Design of New Caffeine Analogues Molecules with Potential Epithelial Anticancer Activity. Curr. Pharm. Des..

[102] Gey van Pittius N.C., Sampson S.L., Lee H., Kim Y., van Helden P.D., Warren R.M. (2006). Evolution and expansion of the *Mycobacterium tuberculosis* PE and PPE multigene families and their association with the duplication of the ESAT-6 (esx) gene cluster regions. BMC Evol. Biol..

[103] Mysinger M.M., Carchia M., Irwin J.J., Shoichet B.K. (2012). Directory of useful decoys, enhanced (DUD-E): Better ligands and decoys for better benchmarking. J. Med. Chem..

[104] DeLong E.R., DeLong D.M., Clarke-Pearson D.L. (1988). Comparing the areas under two or more correlated receiver operating characteristic curves: A nonparametric approach. Biometrics.

