# Supplementary material for: In Silico Screening of Isocitrate Lyase for Novel Anti-Buruli Ulcer Natural Products Originating from Africa
Source: Molecules. 2018 Jun 27;23(7):1550. doi: 10.3390/molecules23071550 (PMC6100440; doi:10.3390/molecules23071550)
Supplement: Supplementary file 1 [file molecules-23-01550-s001.zip › Supplementary_Figures_update_1.pdf]

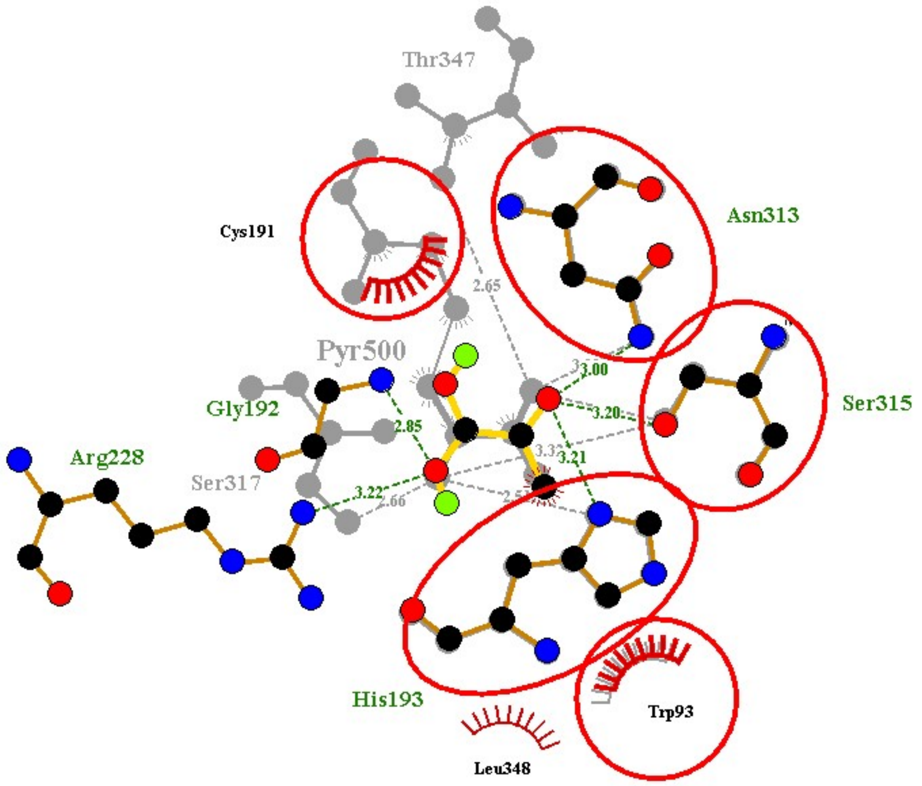

Figure S1: Superimposed Ligplots comparing the interactions between the co-crystalized ligand of 1F8M and the re-docked pyruvic ligand. Residues circled in red color are predicted to bind to the ligand in both the co-crystalized and re-docked complex.

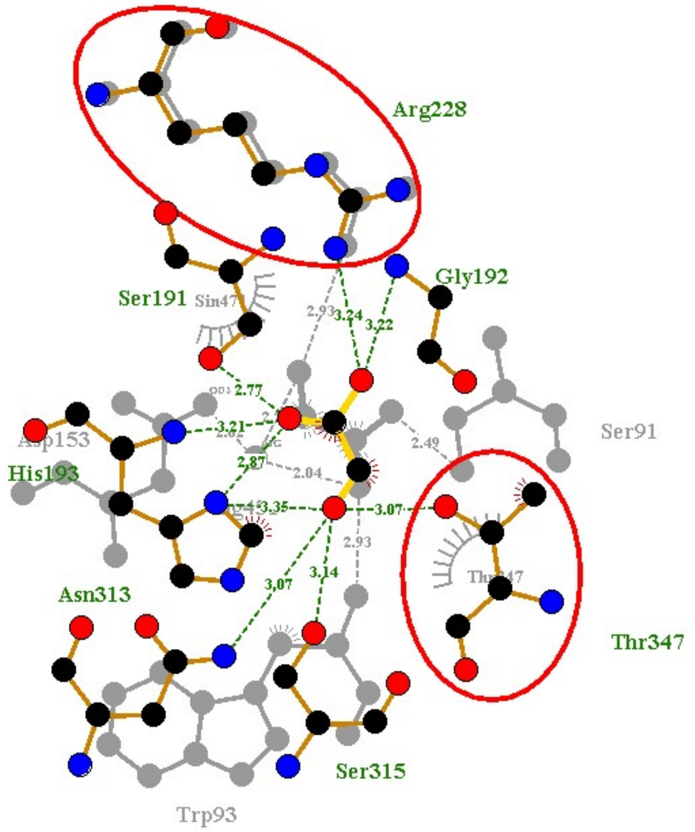

Figure S2: Superimposed Ligplots comparing the interactions between the co-crystalized ligand of 1F8I and the re-docked Glyoxylic acid ligand. Residues circled in red color are predicted to bind to the ligand in both the co-crystalized and re-docked complex

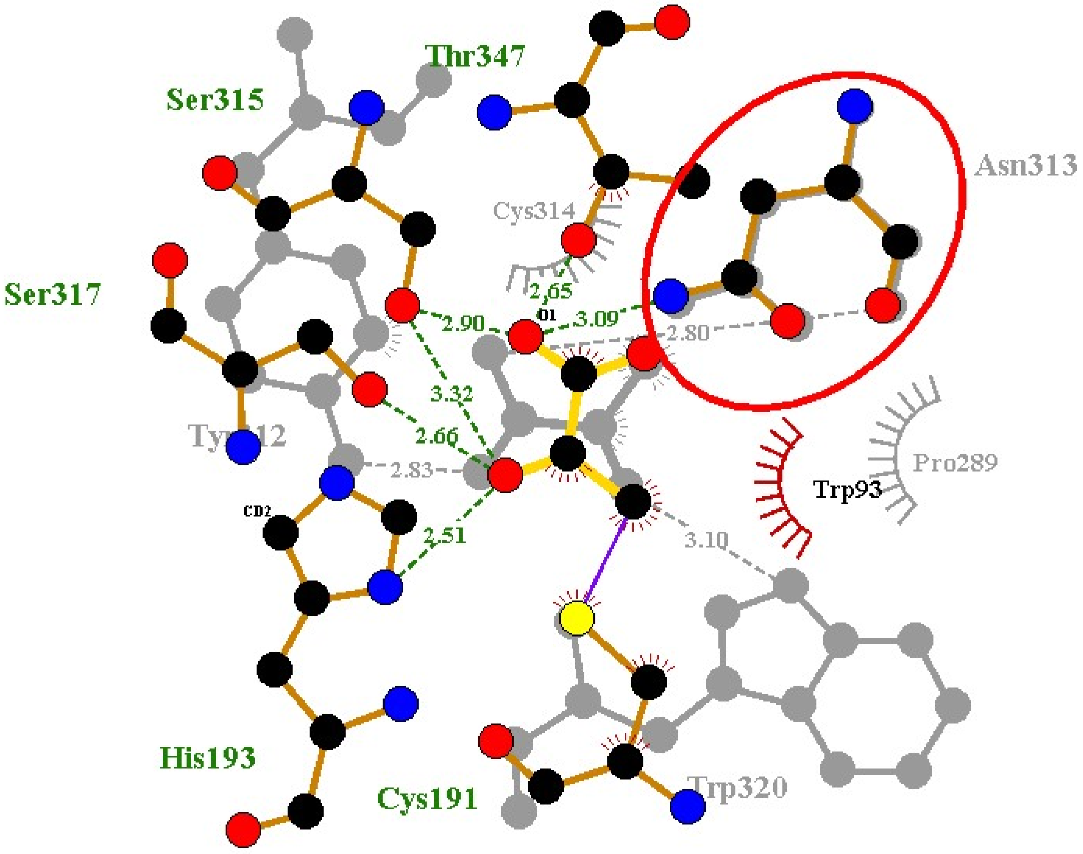

Figure S3: Superimposed Ligplots comparing the interactions between the co-crystalized ligand of 1F8M and the re-docked Pyruvic acid ligand to *M. ulcerans* model. Residues circled in red color are predicted to bind to the ligand in both the co-crystalized and re-docked complex.

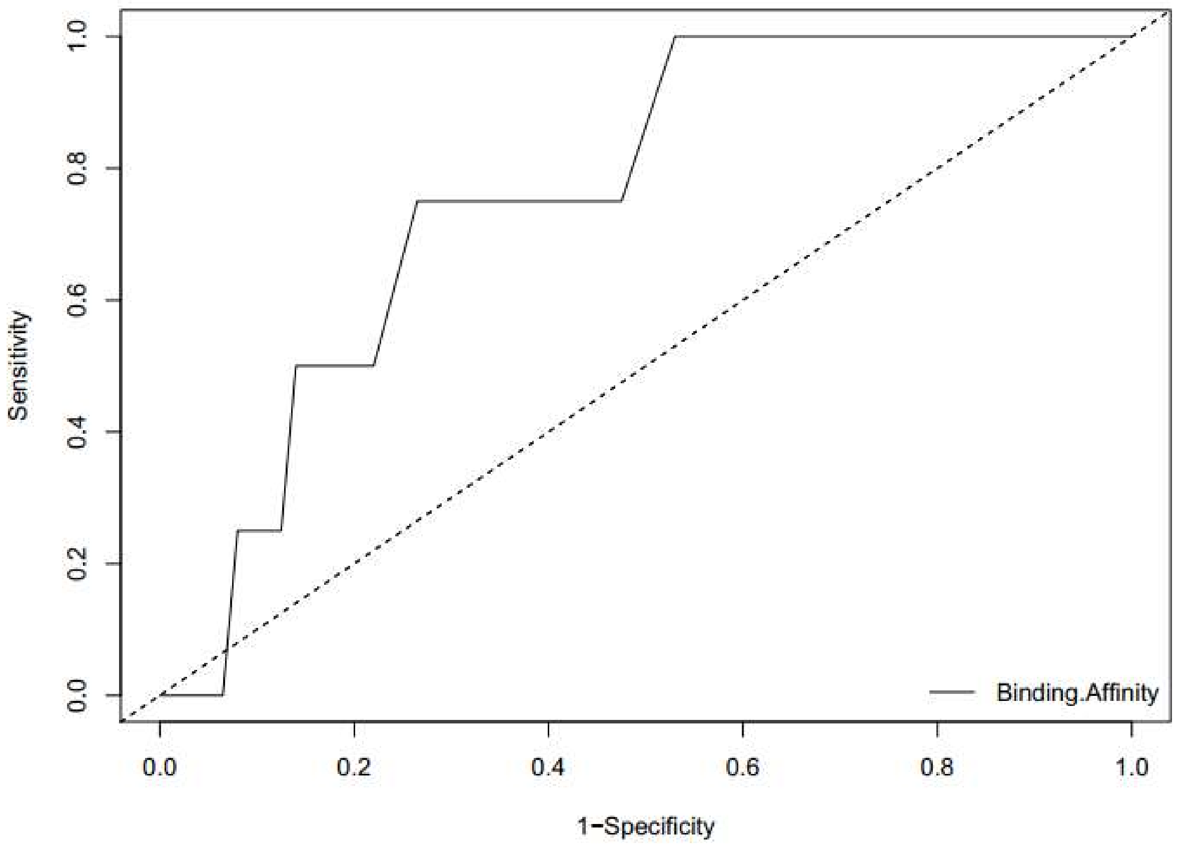

Figure S4: An ROC curve generated by screening co-crystalized ligands from ICL of *M. tuberculosis* with corresponding decoys against the 1F8I of *M. tuberculosis*. The AUC of the ROC curve is 07625, which is considered reasonably good.

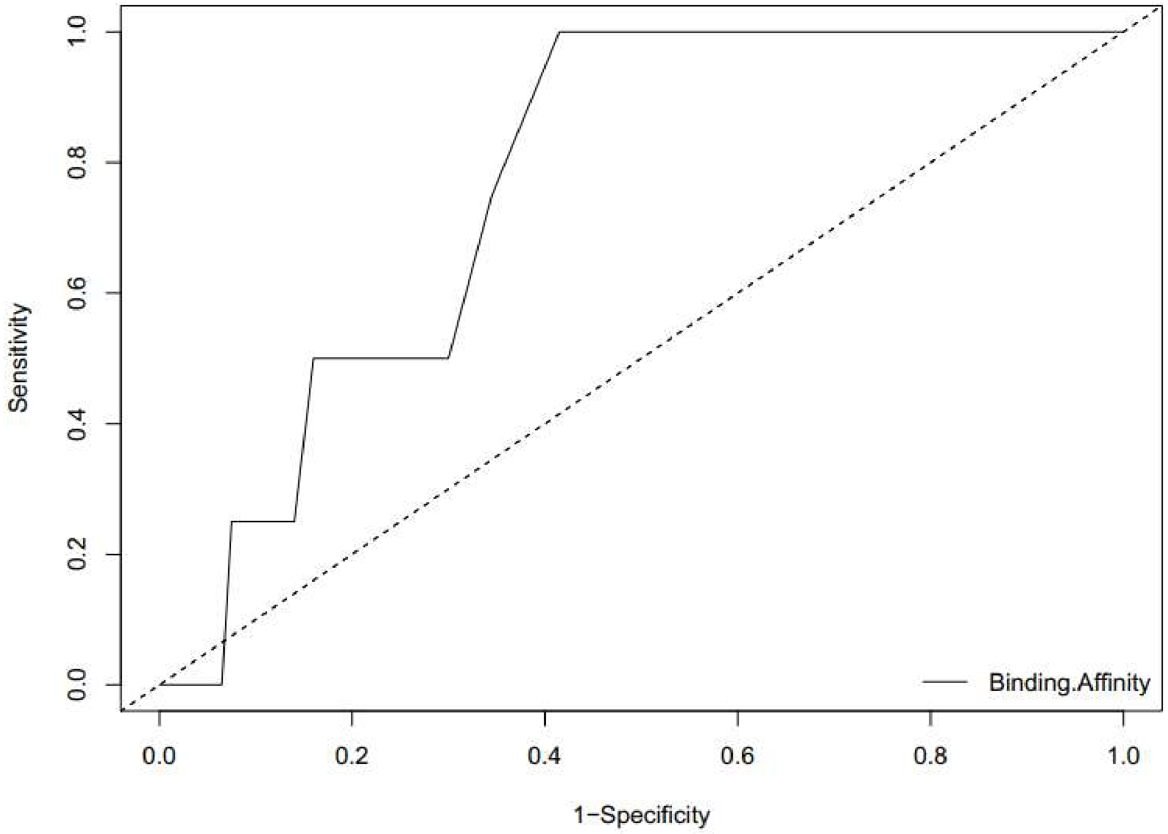

Figure S5: An ROC curve generated by screening co-crystalized ligands from ICL of *M. tuberculosis* with corresponding decoys against the model structure of 1F8M of *M. tuberculosis*. The AUC of the ROC curve is 0.76938, which is considered reasonably good.

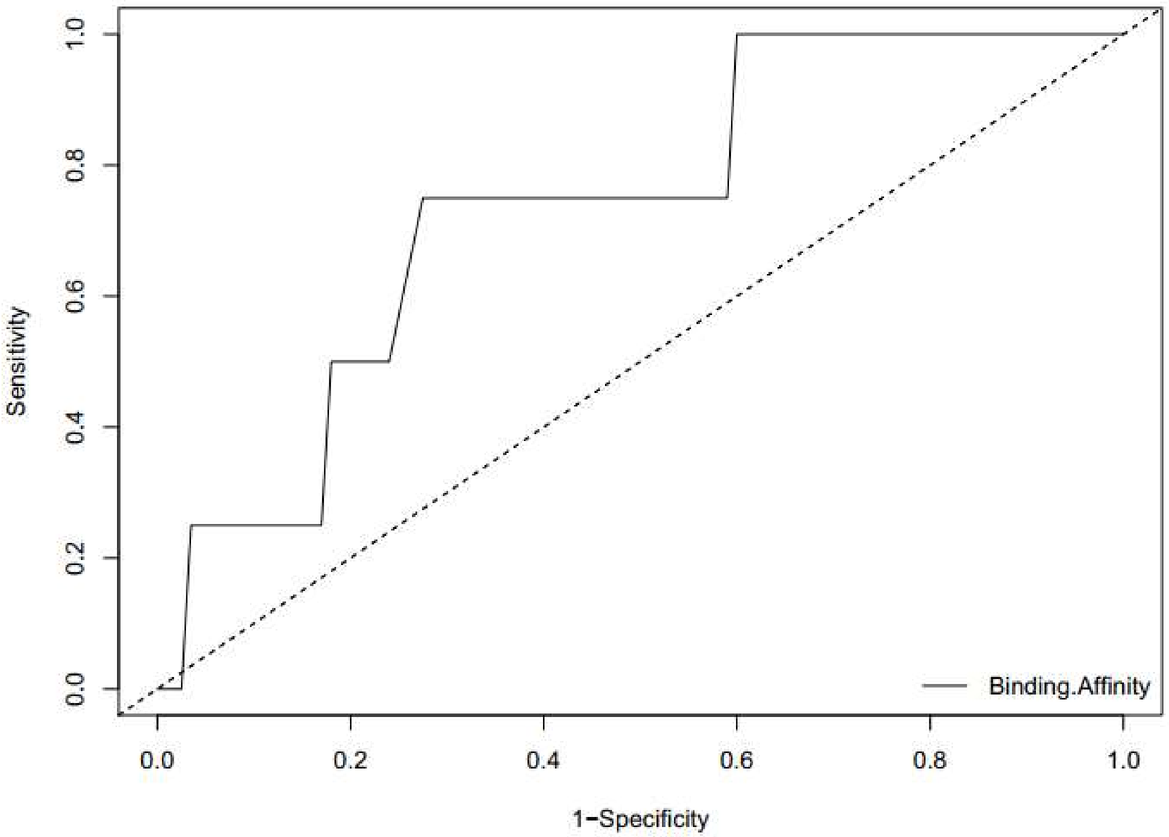

Figure S6: An ROC curve generated by screening co-crystalized ligands from ICL of *M. tuberculosis* with corresponding decoys against the model structure of 5DQL of *M. tuberculosis*. The AUC of the ROC curve is 0.73567, which is considered reasonably good.

(A)

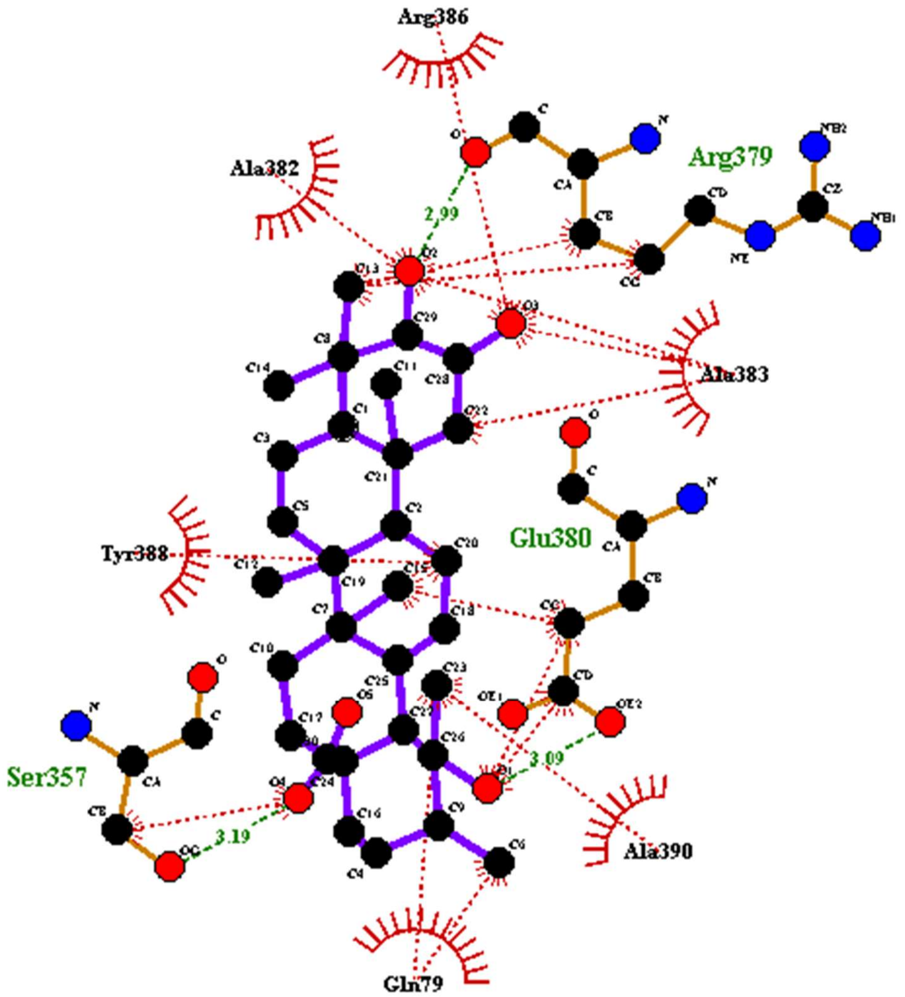
Arg386
Ala382
Arg379
2.99
Ala383
Glu380
Tyr388
Ser357
3.19
3.09
Ala390
Gln79

(B)

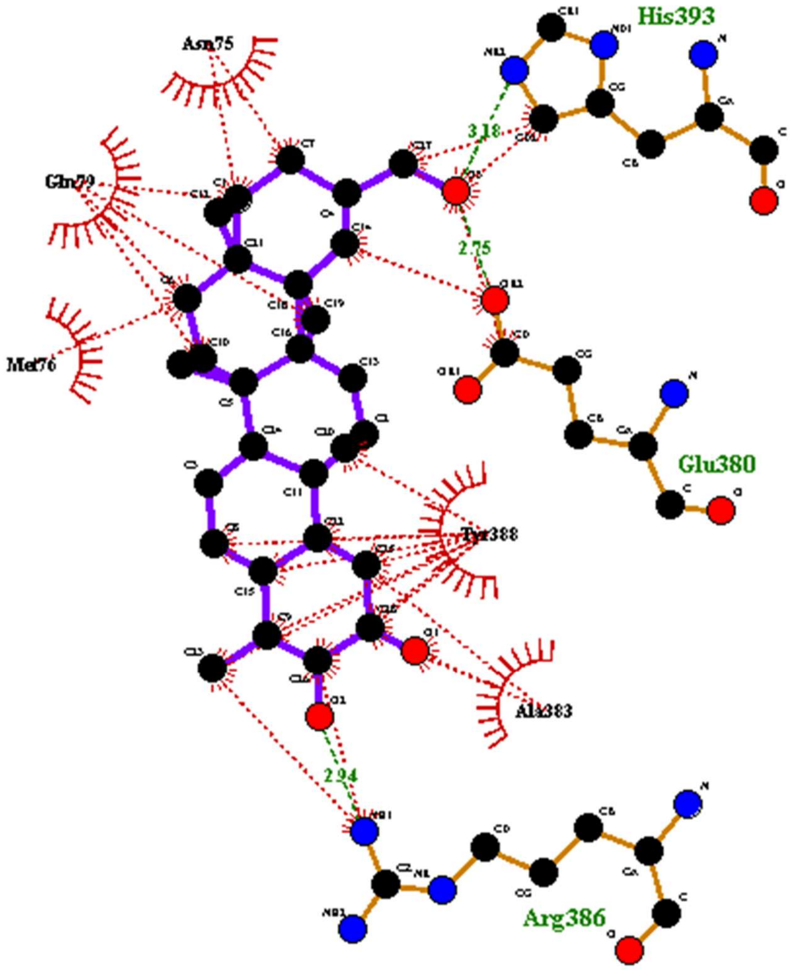

Figure S7. Docking studies and Ligplot+ analysis of Lead molecules. (**A**) Ligplot diagram of ZINC38143792 ligand. Compound interacts via H-Bond with residues Ser357, Arg379, and Glu380. (**B**) Ligplot diagram showing ZINC95485880 interacting with residues Glu380, Arg386 and His393.

(A)

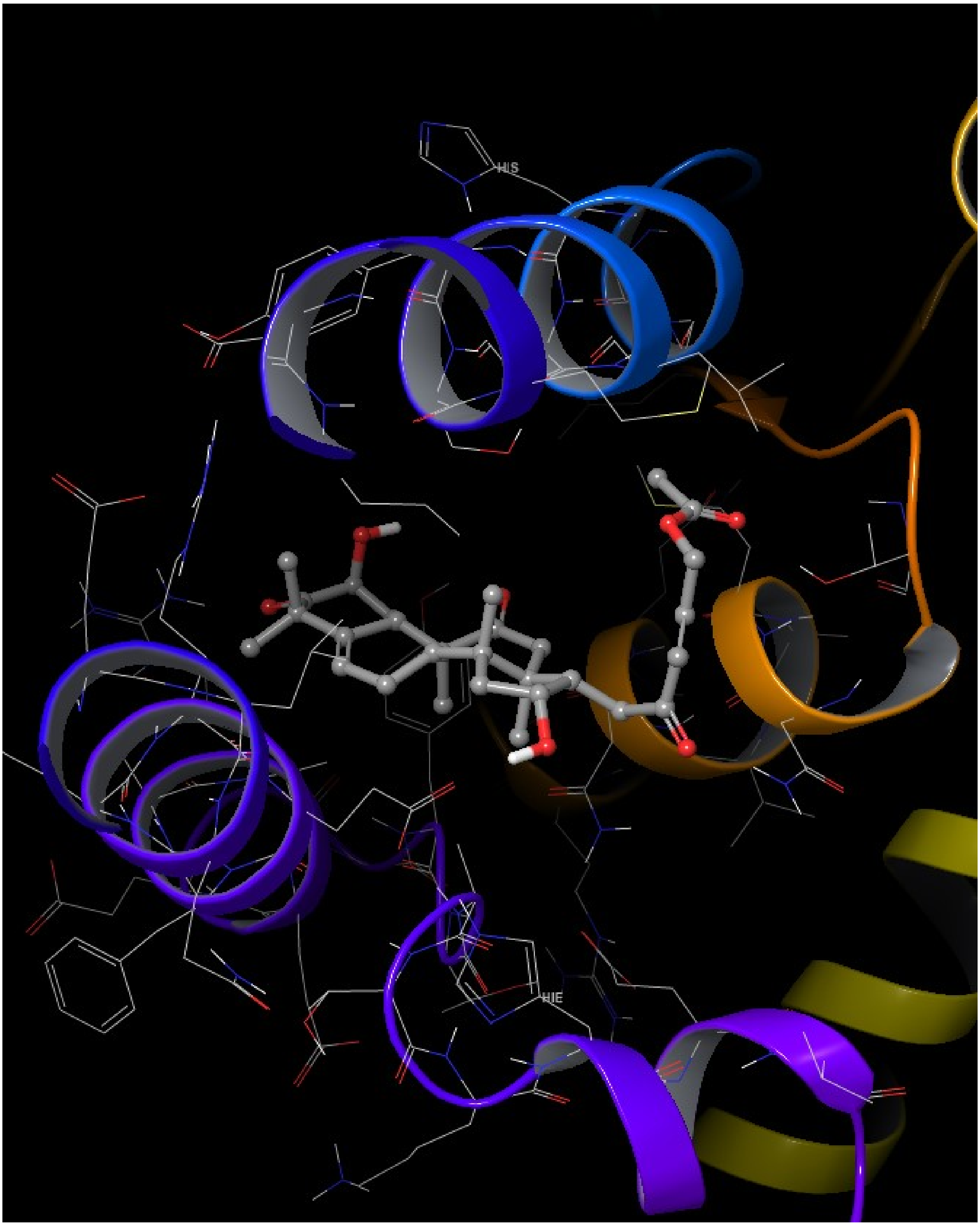

(B)

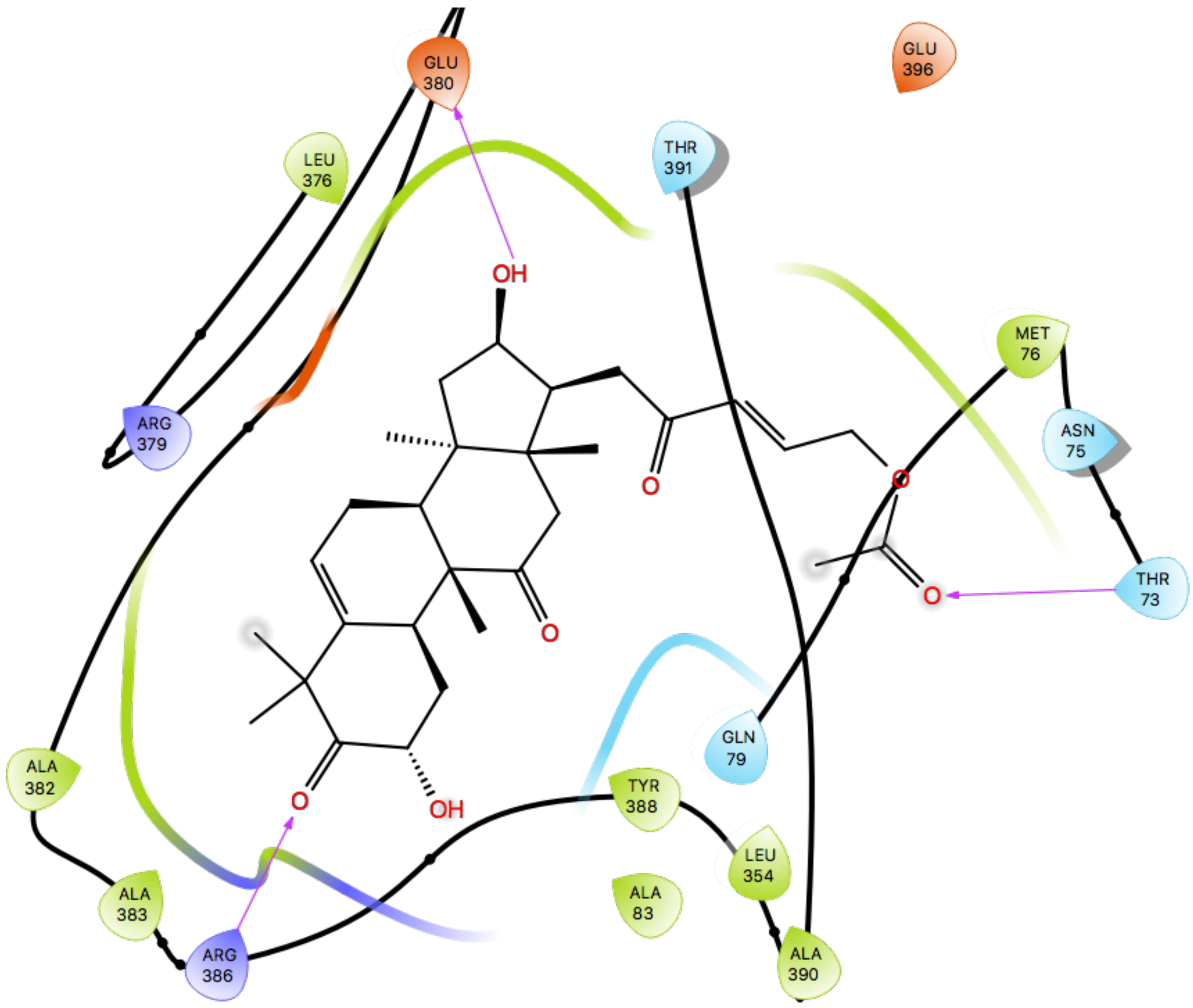

Figure S8: Induced-fit docking studies of ZINC95486305 ligand complex. The figure illustrates induced fit pose of ZINC95486305 (shades of gray) docked in the active site (A) and purple arrows represent hydrogen bonds (B).

(A)

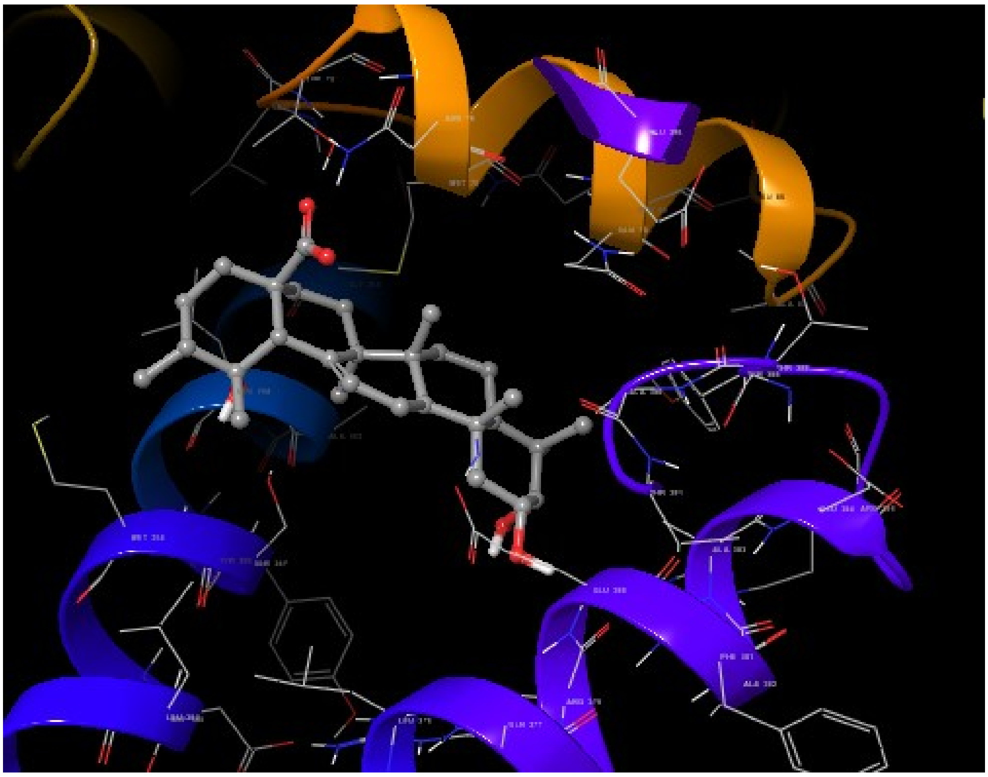

(B)

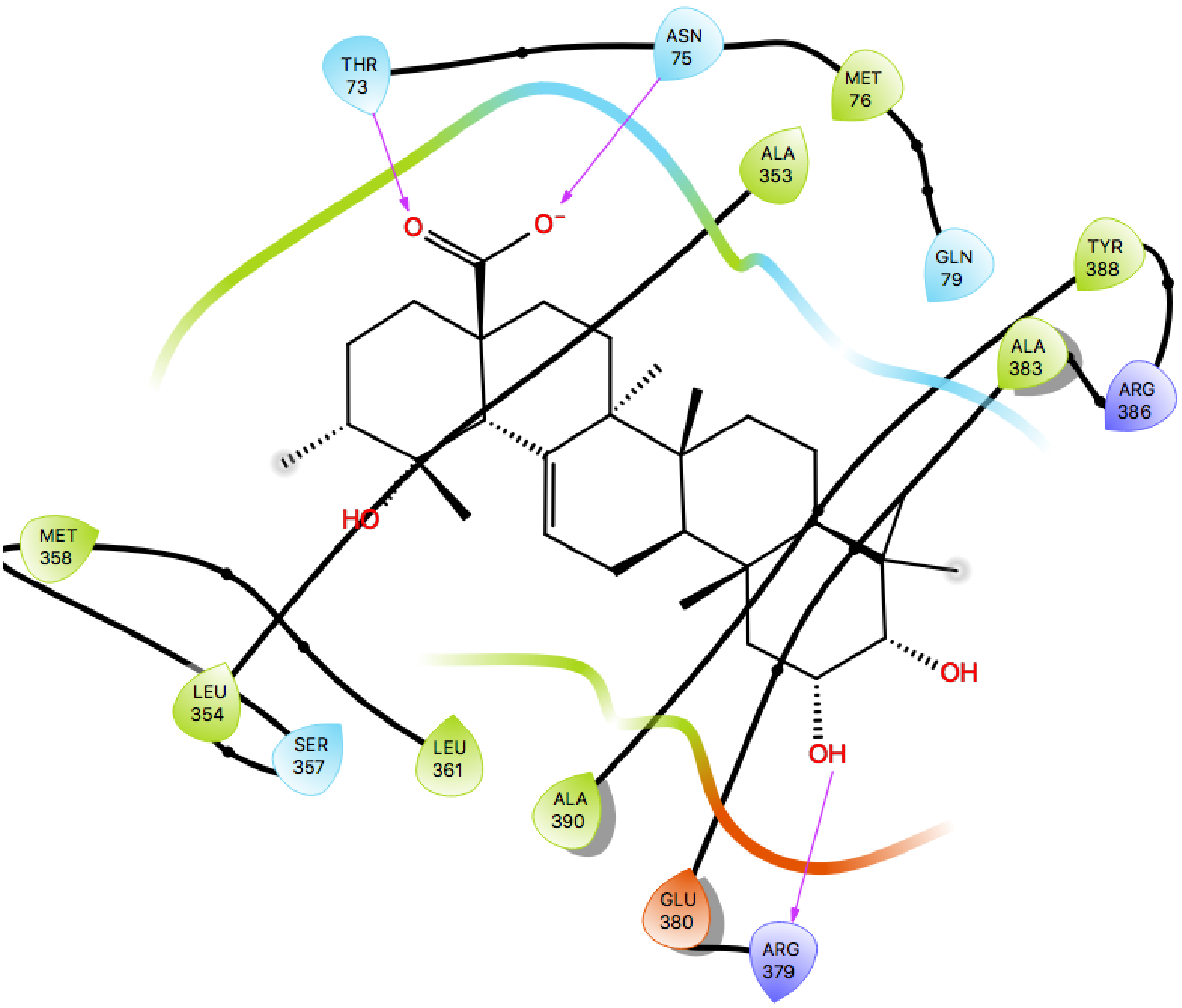

Figure S9: Induced-fit docking studies of ZINC38143792 ligand complex. The figure illustrates the induced fit pose of ZINC38143792 (shades of gray) docked in the active site of ICL (A) and purple arrows represent hydrogen bonds (B).
