# Supplementary material for: In Silico Screening of Isocitrate Lyase for Novel Anti-Buruli Ulcer Natural Products Originating from Africa
Source: Molecules. 2018 Jun 27;23(7):1550. doi: 10.3390/molecules23071550 (PMC6100440; doi:10.3390/molecules23071550)
Supplement: Supplementary file 1 [file molecules-23-01550-s001.zip › Supplementary_Tables_update.pdf]

Molecules **2018**, 23, 1550; doi:10.3390/molecules23071550 www.mdpi.com/journal/molecules

Table S1: A table showing the two-dimensional (2D) structures of 20 selected ligands generated with DrugBank (https://www.drugbank.ca/).

ZINC ID	2D STRUCTURE
ZINC95486006	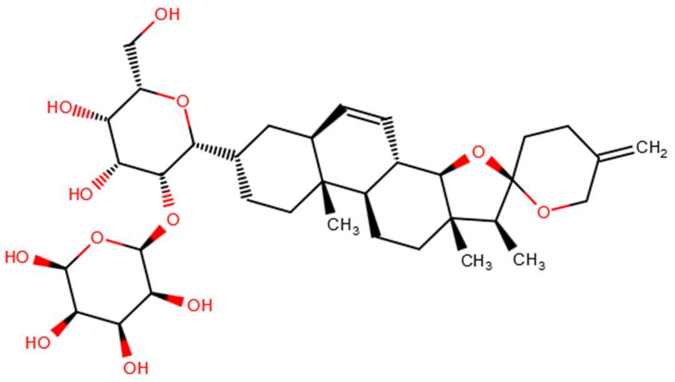
ZINC95486007	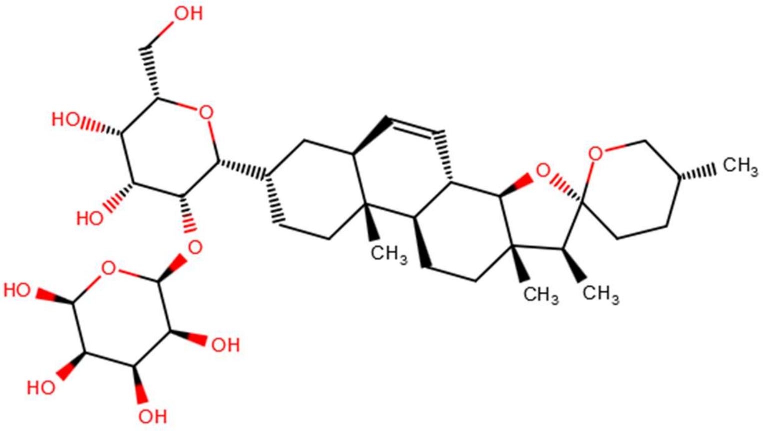
ZINC38143792	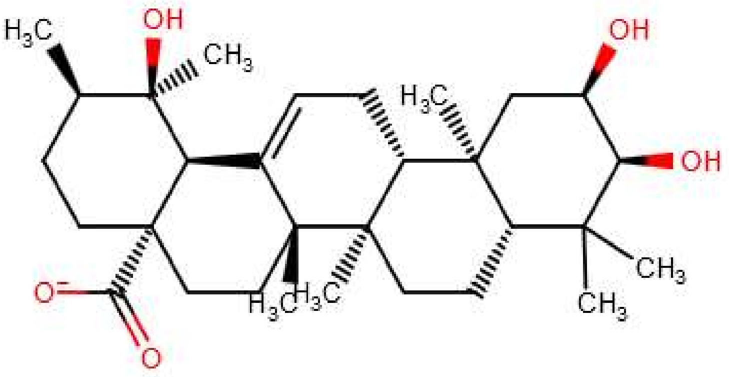

ZINC95485880	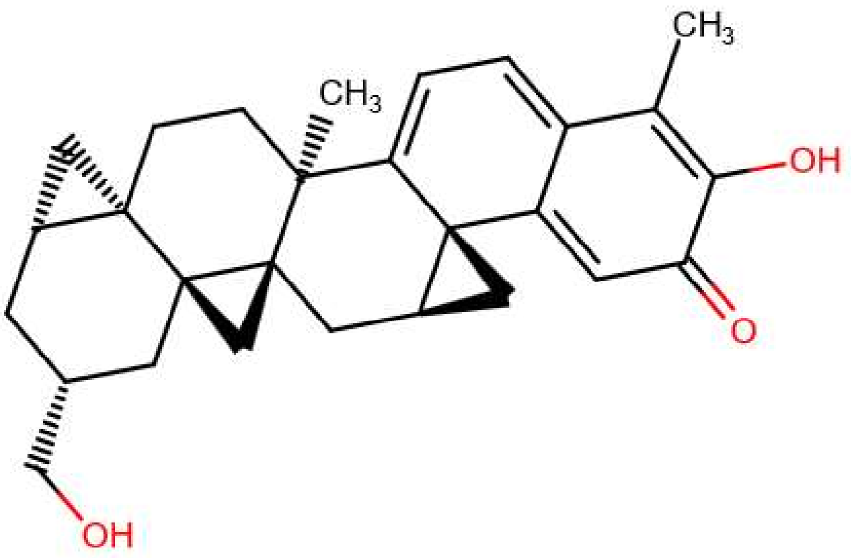
ZINC95486305	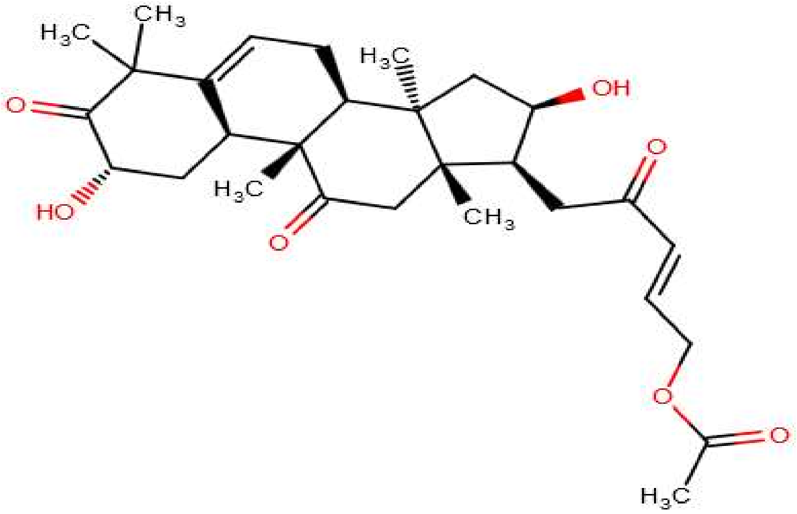

ZINC95486303	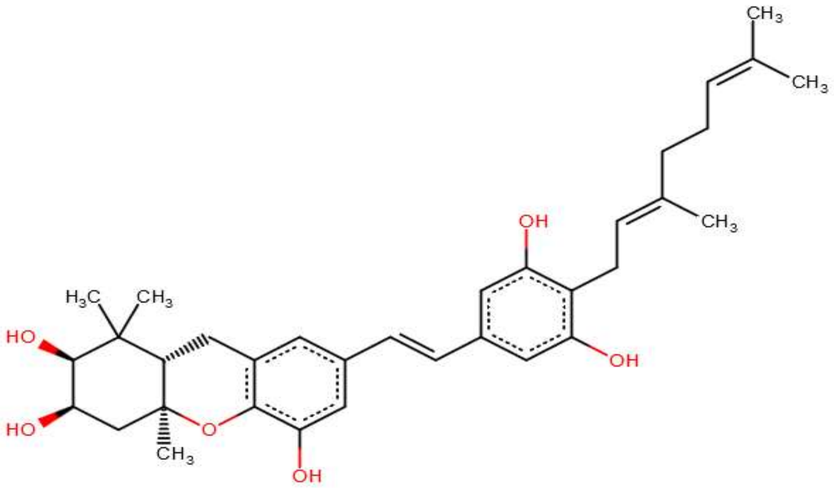
ZINC95485905	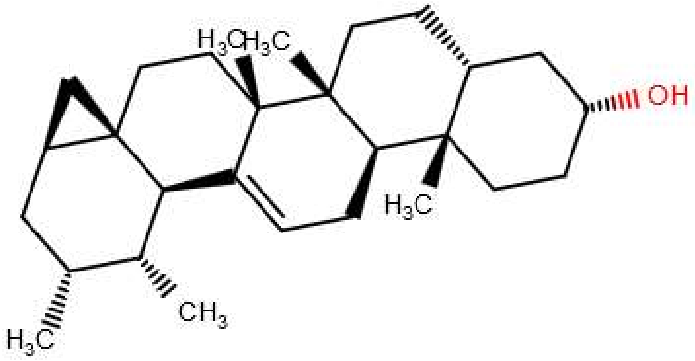
ZINC95486183	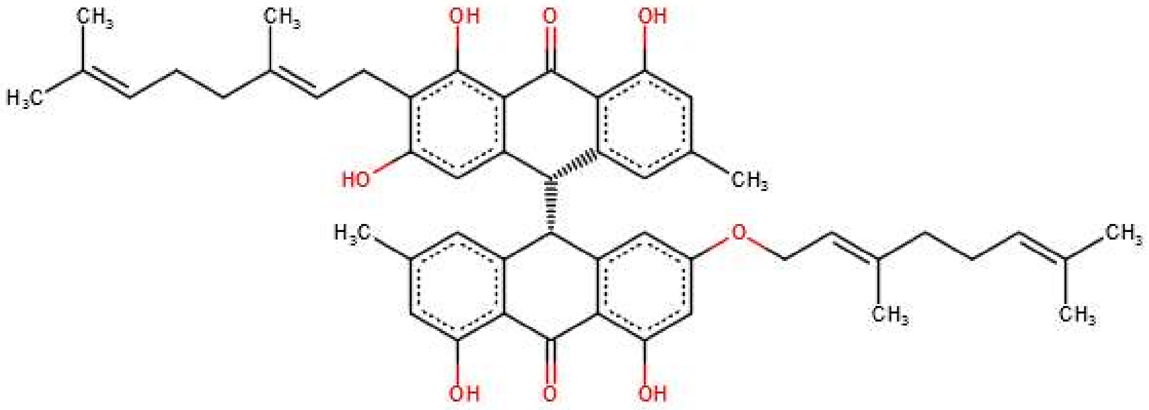

ZINC95486184	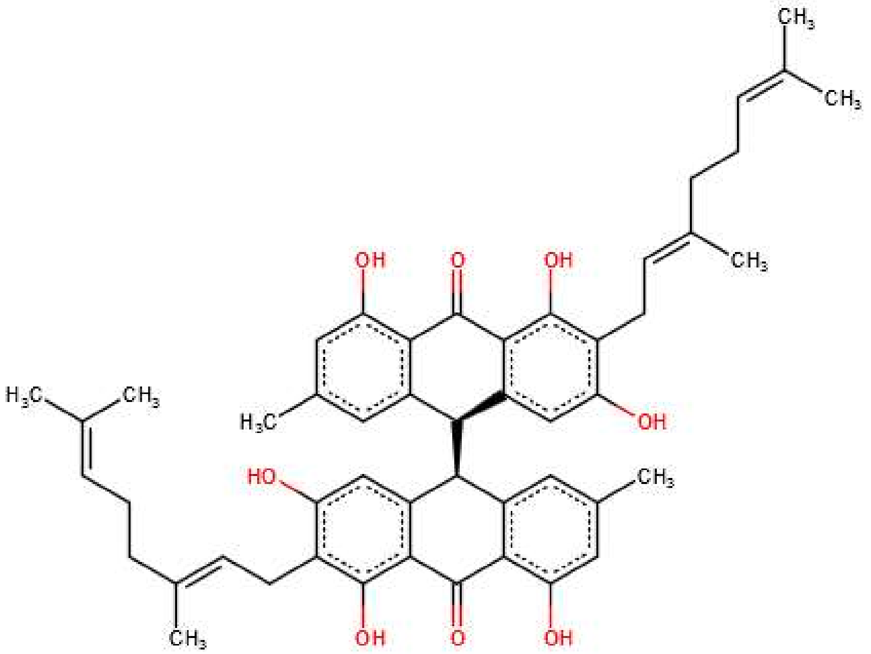
ZINC95486142	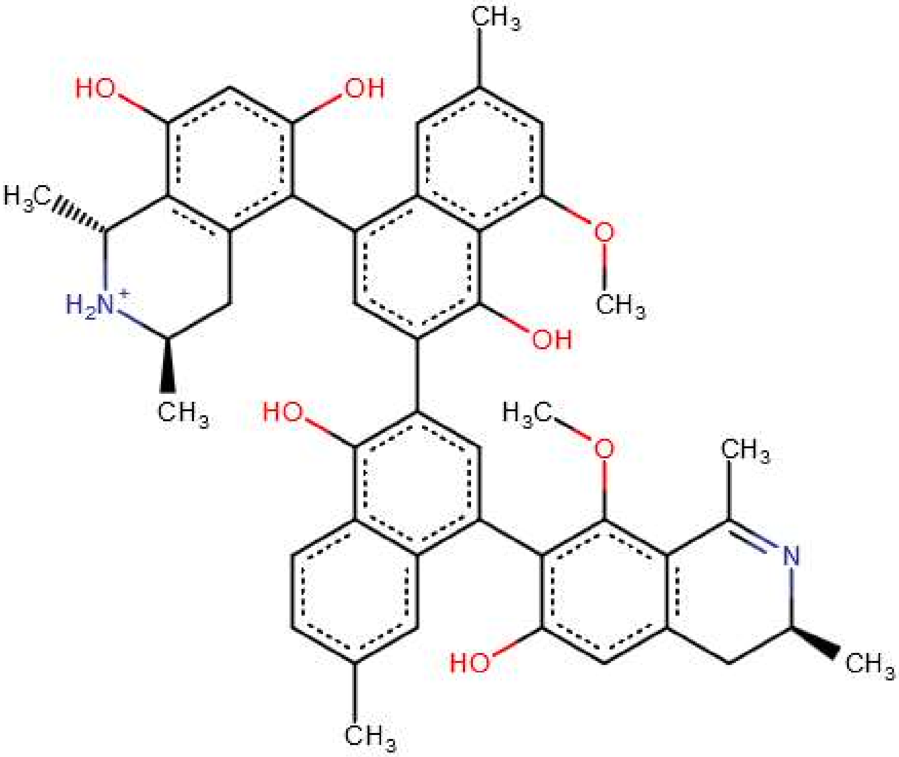

ZINC86037206	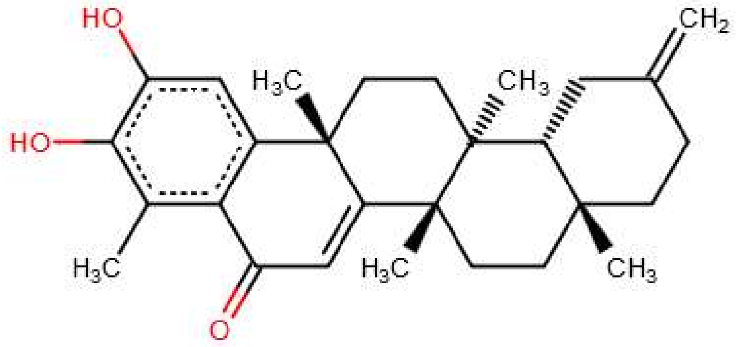
ZINC31761332	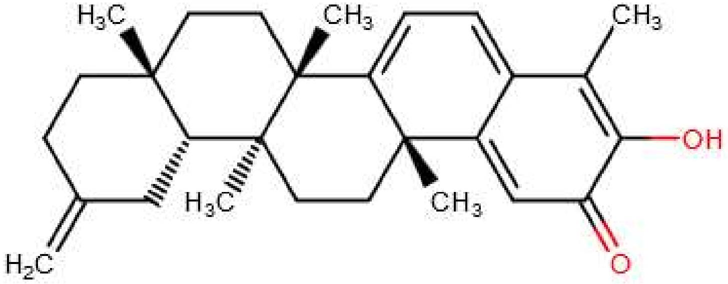
ZINC95486231	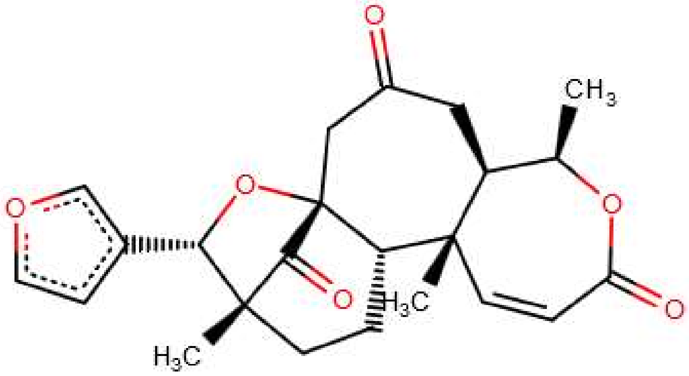
ZINC03197457	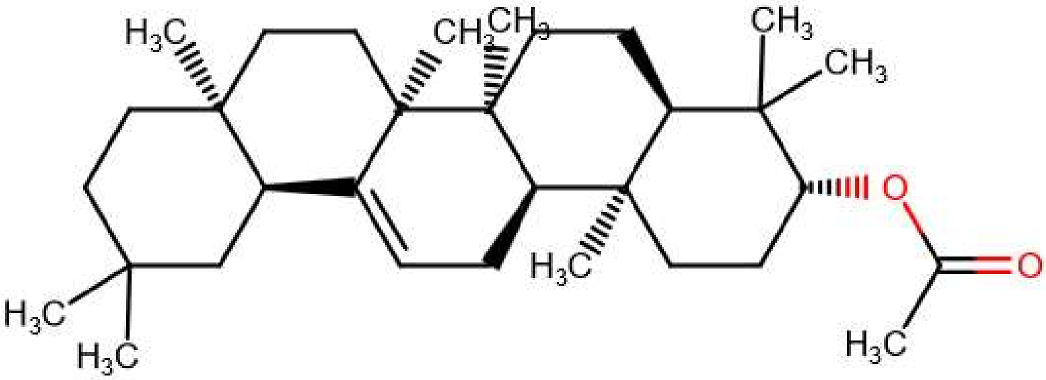

ZINC95485943	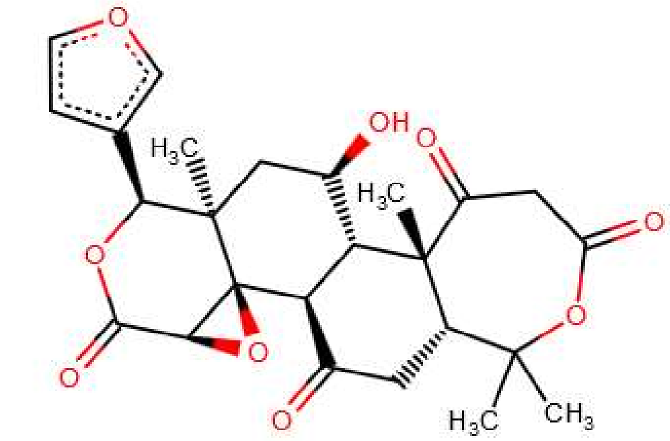
ZINC95486001	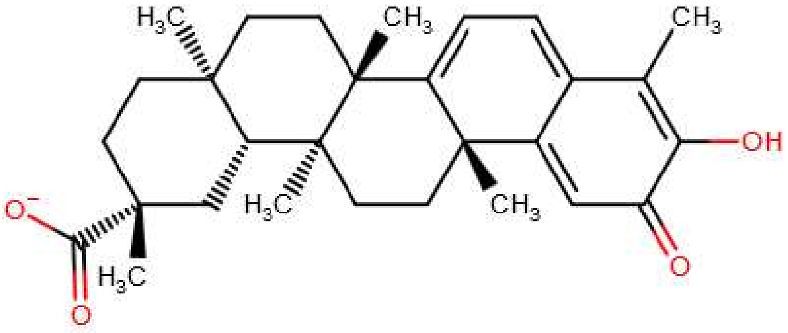
ZINC40431237	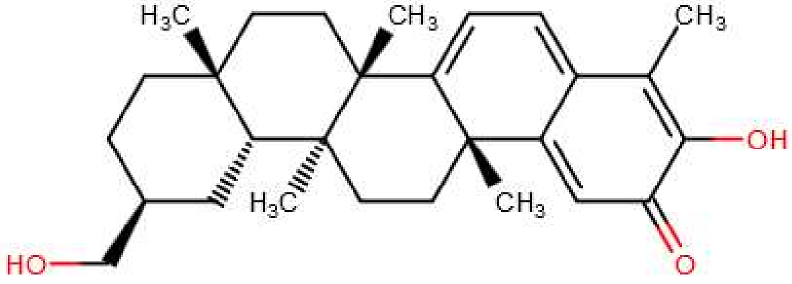

ZINC95486182	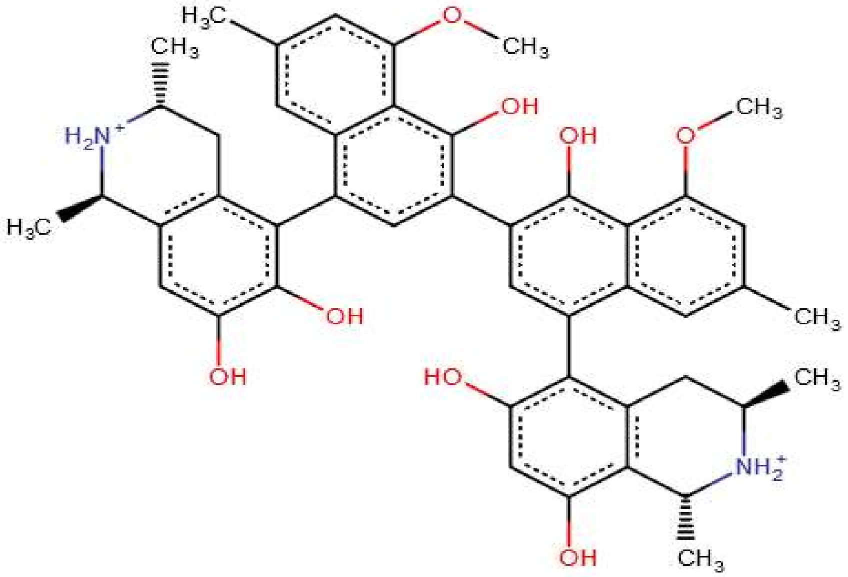
ZINC03941105	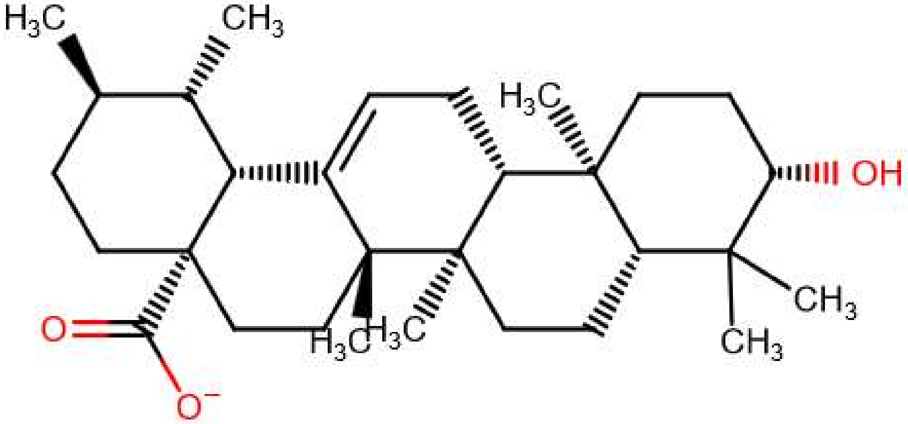

ZINC95485882	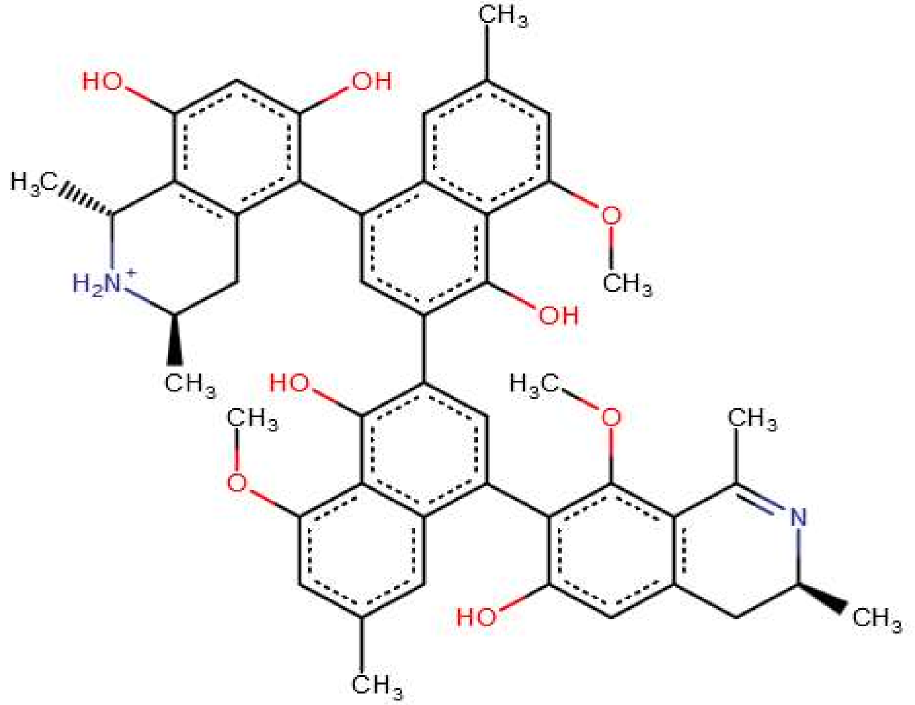

Table S2: A table showing the 20 selected ligands. The binding energies of the ligands are shown in kcal/mol. Also shown are the residues within the active site of the ICL protein that are interacting with the ligands via hydrogen bonding and hydrophobic interactions.

**Predicted Ligands**	**Binding Energy/ (Kcal/mol)**	**Hydrogen bond interacting residues**	**Hydrophobic bond interacting residues**
ZINC95486006	-9.5	Asn75, Ser357, Glu380, Ala390	Met76,Gln79,Ala353, Leu354,Met358,Leu361,Ala362, Tyr365,Tyr373, Leu376,His393, Glu396
ZINC95486007	-8.7	Glu380, Asn75, Ala390	Met76, Gln79, Leu354, Ser357, Met358, Leu361, Ala362, Tyr365 Tyr373, Leu376, His393, Glu396
ZINC38143792	-8.6	Glu380 Arg379 Ser357	Gln79, Ala382 , Ala383, Arg386, Tyr388, Ala390
ZINC95485880	-8.6	Glu380 Arg386 His393	Asn75, Met76, Gln79, Ala383, Tyr388
ZINC95486305	-8.8	Gln79, Arg379	Asn75, Glu396, His393, Ala390, Glu380, Tyr388, Arg386, Ala382, Ala383

ZINC95486303	-8.7	Asn319, Lys321	Asn67, Leu69, Gln79, Gln80, Ala83, Leu85, Pro316, Trp320, Ile329, Ile346, Ala349, Ala353, Tyr388
ZINC95485905	-8.5	Glu380 His393	Asn75, Gln79, Leu376, Arg379, Ala383, Tyr388, Ala390
ZINC95486183	-10.0	Glu380	Leu69,Met76,Asn75,Gln79,Pro316, Trp320,Ile346,Ala349,Ala353,Leu376 Trp388,Ala390,His393,Glu396,Val397
ZINC95486184	-9.6	Ala349	Leu69,Asn75,Met76,Gln79, Pro316,Trp320,Ile346, Gly350,His352,Ala353,Leu354, Ser357, Tyr388
ZINC95486142	-9.4	Pro316	Ser315, Ser317, Asn319, Trp320, Lys321, Ile346, Ala349, His352, Asn355
ZINC86037206	-9.3	Asn75	Met76, Gln79, Leu354, Arg379, Glu380, Ala383, Tyr388, Ala390
ZINC31761332	-9.2	Asn75	Met76, Gln79, Leu354, Arg379, Glu380, Ala383, Tyr388, Ala390
ZINC95486231	-9.1	Thr73	Asn75, Met76, Gln79, Leu354, Ser357, Glu380, Ala383, Tyr388, Ala390
ZINC03197457	-8.9	Thr73	Asn75, Met76, Gln79, Leu354, Arg379, Glu380, Ala383, Tyr388, Ala390
ZINC95485943	-8.7	Gln79	Asn75, Met76, Leu354, Leu376, Arg379, Glu380, Ala383, Tyr388, Ala390
ZINC95486001	-8.6	Asn75	Met76, Gln79, Ala353, Tyr356, Tyr388, Ala390
ZINC40431237	-8.5	Asn75	Gln79, Arg379, Glu380, Ala383, Tyr388, Ala390
ZINC95486182	-8.4	His352	Leu69, Met76, Leu354, Ala349, Ala353 Ser357,Leu361,Leu376,Glu380,Ala383 Tyr388, Ala390, Tyr356

ZINC03941105	-8.4	Ser357	Gln79,Leu376,Arg379,Glu380,Ala382, Ala383, Arg386, Tyr388, Ala390
ZINC95485882	-9.3	No residues	Leu69, Gln79, Gln80, Leu85, Ala83, Ile346, Ala349, His352, Ala353, Ser357, Leu376, Arg379,Glu380,Arg386,Tyr388, Ala390

Table S3: Drug-likeness and water solubility of top 20 hits and 5 known drugs (where No. HA = Number of H- bond acceptors, MW = Molecular weight, No. HD = Number of H-bond donors, Bio Sc = Bioavailability Score)

Compound ZINC ID/ Name	Number of Lipinski's Rules Violated	MW (g/mol)	No. HA	No. HD	xLogP	Water Solubility (mg/ml)	Log S	Bio. Sc
ZINC95486006	3	666.805	12	7	0.86	Moderately soluble	-4.46	0.17
ZINC95486007	3	668.821	12	7	1.02	Moderately soluble	-4.86	0.17
ZINC38143792	0	487.701	5	3	4.93	Moderately soluble	-5.92	0.56
ZINC95485880	0	416.561	3	2	3.79	Moderately soluble	-5.03	0.55
ZINC95486305	1	500.362	7	2	2.49	Soluble	-3.72	0.55
ZINC95486303	2	548.72	6	5	8.29	Poorly soluble	-7.73	0.55
ZINC95485905	1	396.569	1	1	7	Poorly soluble	-7.13	0.55
ZINC95486183	2	782.974	8	5	9.85	Insoluble	-12.55	0.17
ZINC95486184	3	782.974	8	6	9.82	Insoluble	-12.68	0.17
ZINC95486142	3	739.889	9	7	9.14	Poorly soluble	-9.91	0.17
ZINC86037206	1	420.593	3	2	6.21	Poorly soluble	-6.97	0.55
ZINC31761332	1	404.594	2	1	5.78	Poorly soluble	-6.33	0.55
ZINC95486231	0	398.455	6	0	2.46	Soluble	-3.73	0.55
ZINC03197457	1	468.766	2	0	8.55	Poorly soluble	-8.74	0.55
ZINC95485943	0	472.49	9	1	1.25	Soluble	-3.30	0.55
ZINC95486001	1	449.61	4	1	5.06	Poorly Soluble	-6.30	0.56
ZINC40431237	0	422.609	3	2	4.94	Moderately soluble	-5.93	0.55
ZINC95486182	3	758.912	10	10	8.89	Poorly soluble	-9.88	0.17
ZINC03941105	1	455.703	3	1	6.79	Poorly soluble	-7.22	0.56

ZINC95485882	3	769.915	10	7	9.13	Insoluble	-10	0.17
RIFAMPICIN	3	822.94	14	6	3.07	Poorly soluble	-8.18	0.17
STREPTOMYCIN	3	581.57	15	11	-5.83	Soluble	1.80	0.17
CLARITHROMYCIN	2	747.95	14	4	2.13	Moderately soluble	-5.94	0.17
MOXIFLOXACIN	0	401.43	6	3	1.85	Soluble	-2.70	0.55
AMIKACIN	3	585.60	17	13	-5.91	Highly Soluble	2.23	0.17
